# Supplementary material for: Improving cell-specific recombination using AAV vectors in the murine CNS by capsid and expression cassette optimization
Source: Mol Ther Methods Clin Dev. 2024 Jan 10;32(1):101185. doi: 10.1016/j.omtm.2024.101185 (PMC10811426; doi:10.1016/j.omtm.2024.101185)
Supplement: Document S1. Figures S1–S5 and Tables S1 and S2 [file mmc1.pdf]

OMTM, Volume *32*

Supplemental information

Improving cell-specific recombination using AAV vectors in the murine CNS by capsid and expression cassette optimization

Hayato Kawabata, Ayumu Konno, Yasunori Matsuzaki, Yumika Sato, Mika Kawachi, Ryo Aoki, Saki Tsutsumi, Shota Togai, Ryosuke Kobayashi, Takuro Horii, Izuho Hatada, and Hirokazu Hirai

mGfaABC1D promoter: 582bp

5’- AACAAATACCATGTCGCTGGTATGGAGTATAGGCTGTTGCTATGACAGGAACT
CAGGGGTCTTAACTGGCTTGAGCGCTGGGAGGGGGCAAGCAGCCAGGCCT
TGTCTGTAAGCTGAAGACCTGGCAGTGCTGAGCTGGTCAGCCCCCAGGACC
TCCTTTTGTGCCCACGAGTGACTCACCTTGGCATAGACATAATGGTCAGGGG
TGGGCACGCAGCCTGCTTCCCGCTGTGCTCCAGGCCTCCTTCGATGCTTTC
CGAGAAGTCTATTGAGCTGGGAGCTTGTACTGCACCCGGGGCTGACATCCT
GGCATCCTGGGATAAAAGCAGCCCACGGGGCTGCCCTTGCCATATGCCTCA
CTGGCGGCAGAGAACAAGGCTCTATTCAGCGAGTACCCTGGGGAGTTCTCC
CCCTAGCTGGGCTGGGGCCCAGCTCCACCCCCTCAGGCTATTCAATGGGG
GTGCTTCCAGGAAGTCAGGGGCAGATTTAGTCCAACCCGTTCCTCCATAAAG
GCCCTGACATCCCAGGAGCCAGCAGAGGCAGGGCAGGTTGGAGCGGAGA
CGCATCACCTCTGCGCGCCGC -3’

Figure S1. Sequence of astrocyte-specific mouse GfaABC1D promoter used in this study.

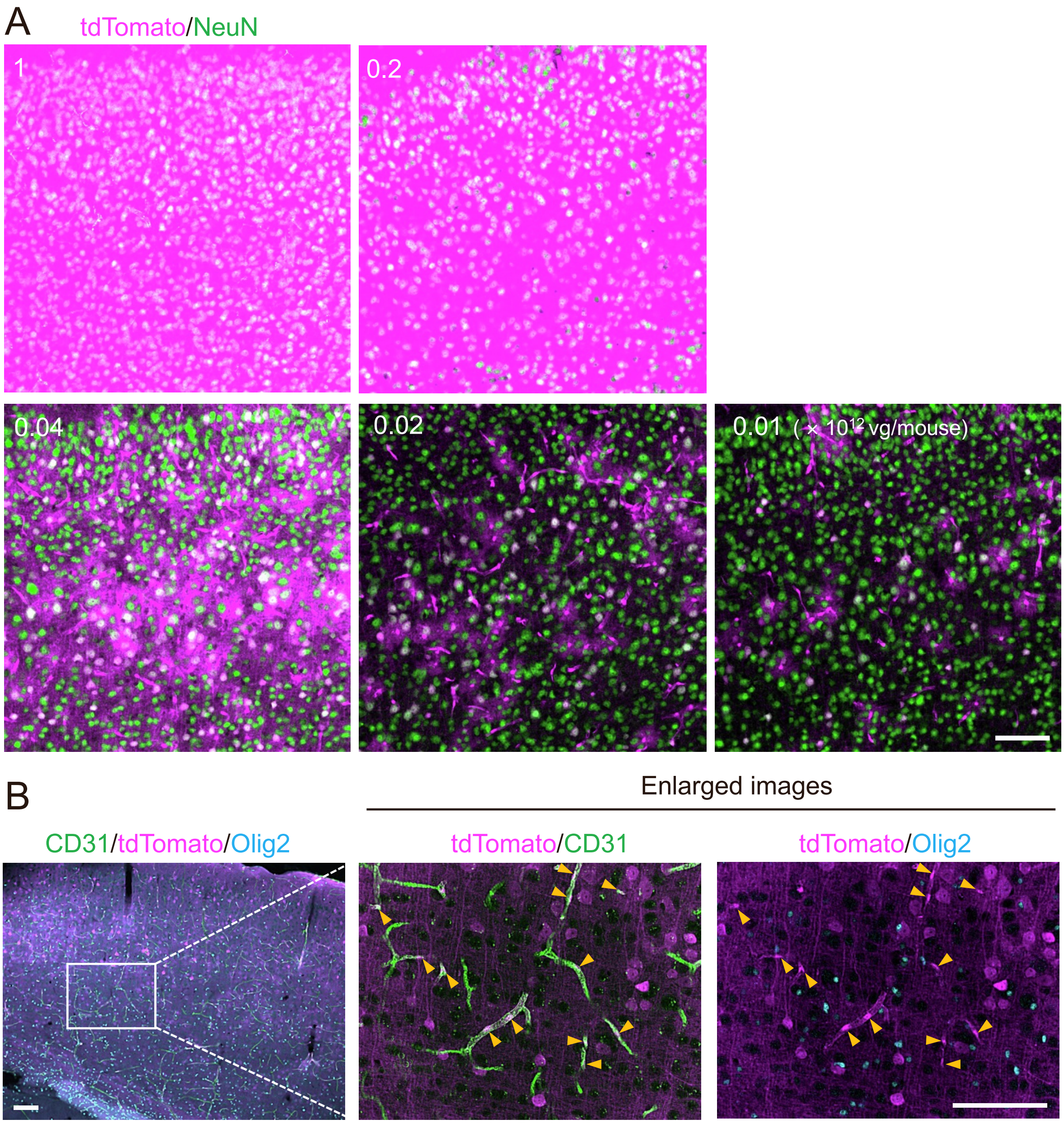

Figure S2. Non-specific cellular transduction in Ai14 floxed mouse brain by intravenously administered PHP.eB expressing Cre using mGfaABC1D promoter. Immunohistochemistry of cerebral cortex three weeks after administration of AAV injection. Mice received injection of AAV vectors serially diluted from 1×10^{12} vg/mouse to 1×10^{10} vg/mouse as shown on the upper left corner of each panel. (**A**) Numerous tdTomato-expressing cells immunolabeled for NSE, a neuron marker. (**B**) Expression of tdTomato in CD31-positive brain microvascular endothelial cells (orange arrowheads). Sagittal brain sections were

immunolabeled for CD31 (green), a marker of brain microvascular endothelial cell and Olig2 (light blue), and a marker of oligodendrocyte. Scale bars; 100 μm.

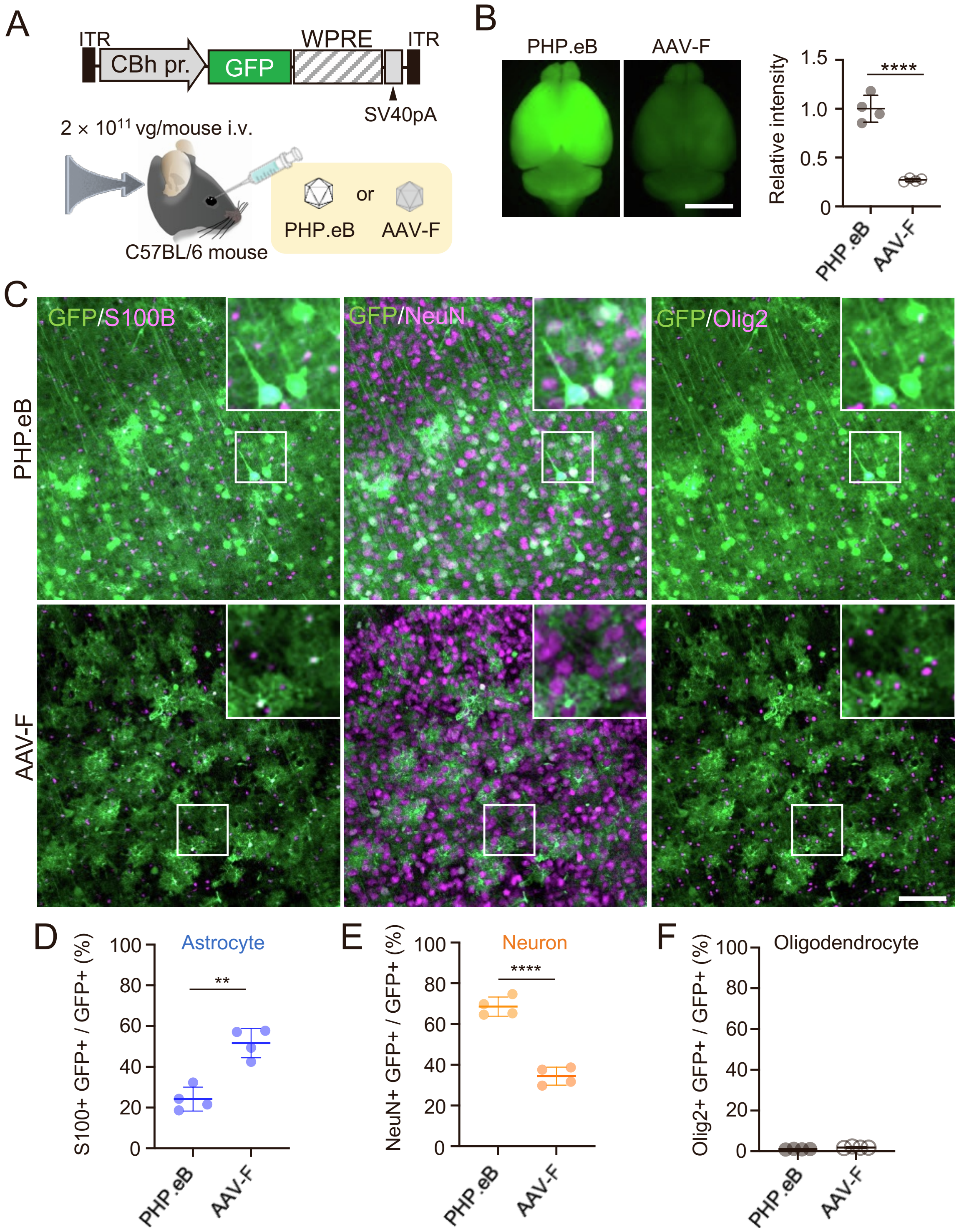

Figure S3. Higher astrocyte-tropic nature of AAV-F over PHP.eB. (**A**) Schematic diagram showing experimental procedure. PHP.eB or AAV-F capsid vectors expressing green fluorescence protein (GFP) under control of ubiquitously active chicken β-actin hybrid

(CBh) promoter were injected into wild-type C57BL/6 mice through orbital plexus. (**B**) Significantly higher fluorescence intensity in PHP.eB-treated mouse brain than in AAV-F-treated brain. GFP fluorescence intensity of whole brains was measured three weeks after AAV injection. Data (average ± SD) were obtained from four mice and value from each mouse was plotted. ****P<0.0001 by unpaired student's *t*-test. Scale bar; 5 mm. (**C**) Triple immunostaining of brain sagittal sections for S100B, NeuN, and Olig2, an oligodendrocyte marker, three weeks after AAV injection. Fluorescence images of primary motor cortex from PHP.eB-treated (upper panels) and AAV-F-treated (lower panels) mice were presented, in which square regions were enlarged and shown in upper right corners. Scale bar; 100 μm. (**D–F**) Graph showing specificity for transduction of astrocytes (**D**), neurons, (**E**) and oligodendrocytes (**F**) in mice treated with PHP.eB or AAV-F vectors. Data (average ± SD) were obtained from four mice and value from each mouse was plotted. **P<0.01 and ***P<0.001 by unpaired student's *t*-test. ITR; inverted terminal repeat, WPRE; woodchuck hepatitis virus post-transcriptional regulatory element, SV40pA; simian virus 40 polyadenylation signal.

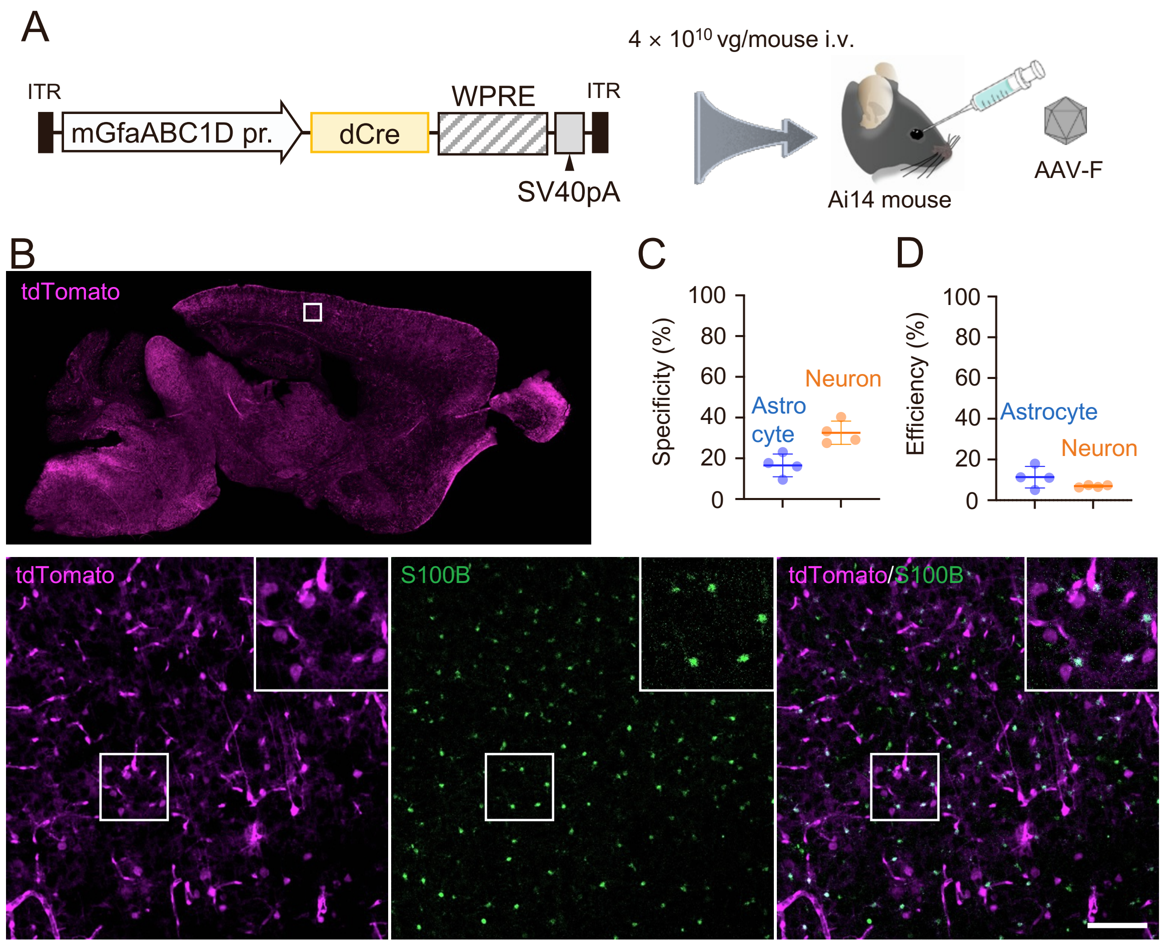

Figure S4. Non-specific recombination in Ai14 mouse brain by AAV-F- mGfaABC1D promoter-dCre. (**A**) Schematic diagram showing experimental procedure. Ai14 mice received intravenous injection of AAV-F-mGfaABC1D promoter-dCre. (**B**) Double immunostaining of brain sagittal sections for S100B (green) and NeuN (not shown) three weeks after AAV injection, showing that most of tdTomato-expressing cells were not immunolabeled for S100B. Scale bar; 100 µm. (**C**, **D**) Graph showing specificity (**C**) and efficiency (**D**) for astrocyte and neuronal transduction. Data (average ± SD) were obtained from four mice and value from each mouse was plotted. The specificity and efficiency for astrocytes are presented in Table S1.

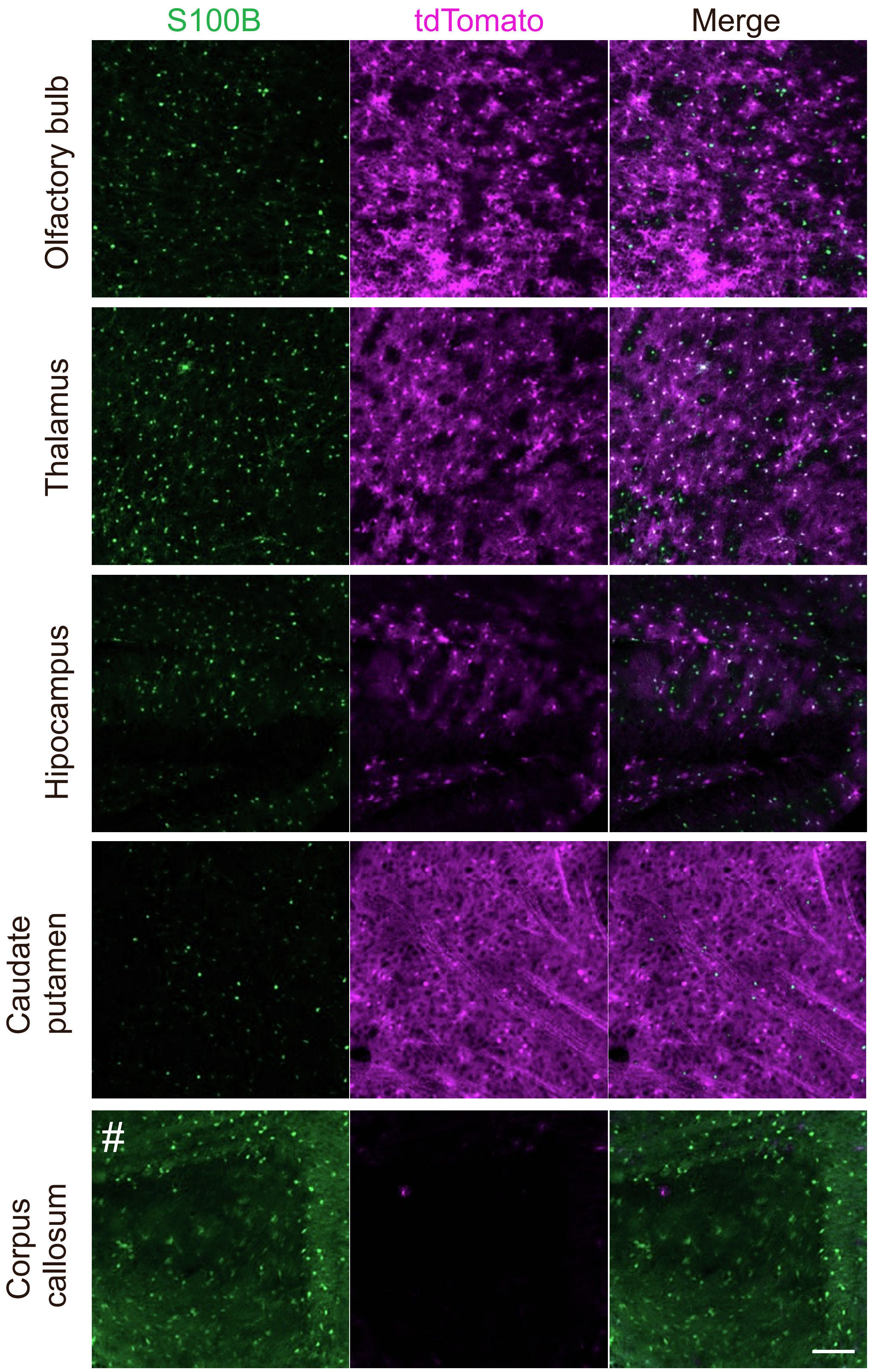

Figure S5. Recombination profiles of various regions in Ai14 mouse treated with AAV-F/FlpO-dCre. Ai14 mice were treated with AAV-F/FlpO-dCre as depicted in Fig. 5A and

examined three weeks after the injection. Fluorescence images of S100B immunolabeling (green, left panels), native tdTomato (magenta, middle panels) and merged images (right panels) from various brain regions. Fluorescent images were obtained using the same parameters except for S100B immunostaining of corpus callosum (#, bottom left panel), which was enhanced because the fluorescence was too weak. Scale bar; 100 μm. The specificity and efficiency of astrocyte recombination in the mentioned brain areas are summarized in Table S2.

Table S1. Summary of the specificity and efficiency of astrocyte transduction in the cerebral cortex by intravenous infusion of PHP.eB or AAV-F capsid vectors. These AAV vectors expressed tdTomato, Cre, FlpO, and dCre under control of mGfaABC1D promoter. Specificity for astrocyte transduction was calculated by dividing number of tdTomato and S100B double-positive cells by number of tdTomato-positive cells. Transduction efficiency was calculated by dividing number of tdTomato and S100B double-positive cells by number of S100B-positive cells. Data (average ± SD) were obtained from four mice.

AAV		Mouse	Dose (vg/mouse)	Astrocyte	
Genome	Capsid			Specificity (%)	Efficiency (%)
mGfaABC1D-tdTomato	PHP.eB	WT	1.0E+12	90.6 ± 4.1	13.2 ± 8.2
mGfaABC1D-Cre	PHP.eB	Ai14	4.0E+10	9.3 ± 3.5	10.8 ± 7.8
mGfaABC1D-Cre	AAV-F	Ai14	4.0E+10	33.6 ± 5.6	17.6 ± 3.6
mGfaABC1D-dCre	AAV-F	Ai14	4.0E+10	16.6 ± 4.8	11.3 ± 4.6
mGfaABC1D-FlpO<br>mGfaABC1D-dCre	PHP.eB	Ai14	5.0E+11<br>5.0E+11	49.9 ± 10.9	43.3 ± 6.8
mGfaABC1D-FlpO<br>mGfaABC1D-dCre	AAV-F	Ai14	5.0E+11<br>5.0E+11	92.6 ± 1.4	42.8 ± 9.7

Table S2. Summary of the specificity and efficiency of astrocyte transduction in different brain regions from Ai14 mice treated with AAV-F/FlpO-dCre. Data (average ± SD) were obtained from four–five mice except for the data on the corpus callosum (n = 2 mice). N.D., not determined.

	Astrocyte	
Region	Specificity (%)	Efficiency (%)
Olfactory bulb	62.0±14.1	48.7±16.3
Thalamus	81.8±15.1	32.2±10.0
Hippocampus	88.4±6.9	22.3±5.7
Caudate putamen	37.3±21.4	8.7±5.9
Corpus callosum	N.D.	N.D.
