# Supplementary material for: Improving cell-specific recombination using AAV vectors in the murine CNS by capsid and expression cassette optimization
Source: Mol Ther Methods Clin Dev. 2024 Jan 10;32(1):101185. doi: 10.1016/j.omtm.2024.101185 (PMC10811426; doi:10.1016/j.omtm.2024.101185)
Supplement: Document S2. Article plus supplemental information [file mmc2.pdf]

Molecular Therapy
Methods & Clinical Development
Original Article

Improving cell-specific recombination using AAV vectors in the murine CNS by capsid and expression cassette optimization

Hayato Kawabata,[1] Ayumu Konno,[1,2] Yasunori Matsuzaki,[1,2] Yumika Sato,[1] Mika Kawachi,[1] Ryo Aoki,[1] Saki Tsutsumi,[1] Shota Togai,[1] Ryosuke Kobayashi,[3] Takuro Horii,[3] Izuho Hatada,[2,3] and Hirokazu Hirai[1,2]

[1]Department of Neurophysiology & Neural Repair, Gunma University Graduate School of Medicine, Maebashi, Gunma 371-8511, Japan; [2]Viral Vector Core, Gunma University, Initiative for Advanced Research, Maebashi, Gunma 371-8511, Japan; [3]Laboratory of Genome Science, Biosignal Genome Resource Center, Institute for Molecular and Cellular Regulation, Gunma University, Maebashi, Gunma 371-8512, Japan

The production of cell-type– and age-specific genetically modified mice is a powerful approach for unraveling unknown gene functions. Here, we present a simple and timesaving method that enables adeno-associated virus (AAV)–mediated cell-type– and age-specific recombination in floxed mice. To achieve astrocyte-specific recombination in floxed Ai14 reporter mice, we intravenously injected blood-brain barrier–penetrating AAV-PHP.eB vectors expressing Cre recombinase (Cre) using the astrocyte-specific mouse glial fibrillary acidic protein (mGfaABC1D) promoter. However, we observed nonspecific neuron-predominant transduction despite the use of an astrocyte-specific promoter. We speculated that subtle but continuous Cre expression in nonastrocytic cells triggers recombination, and that excess production of Cre in astrocytes inhibits recombination by forming Cre-DNA aggregates. Here, we resolved this paradoxical event by dividing a single AAV into two mGfaABC1D-promoter-driven AAV vectors, one expressing codon-optimized flippase (FlpO) and another expressing flippase recognition target–flanked rapidly degrading Cre (dCre), together with switching the neuron-tropic PHP.eB capsid to astrocyte-tropic AAV-F. Moreover, we found that the FlpO-dCre system with a target cell-tropic capsid can also function in neuron-targeting recombination in floxed mice.

INTRODUCTION

Cell-type–specific expression of molecular tools and sensors in the brain is an effective approach for monitoring and regulating the activity and function of target cell types in the nervous system. Conventionally, cell-type–specific transgene expression is performed by randomly inserting a cell-type–specific promoter-transgene cassette into the mouse genome. However, this method makes it difficult to control the level of transgene expression, which can also result in its expression in nontargeted cells. To overcome this limitation, knockin mice have been developed by inserting a transgene at a particular gene locus by homologous recombination. Such knockin mice express a transgene under the control of the endogenous promoter of a target gene, and thus recapitulate the spatiotemporal expression pattern of the target endogenous gene. Crossing targeted knockin Cre recombinase (Cre) driver mice with floxed mice allows recombination only within cells defined by the endogenous promoter of the target gene. Despite these advantages, producing and maintaining multiple mouse lines is expensive and time-consuming.

In recent years, adeno-associated virus (AAV) vectors targeting specific cell populations have been used widely. The application of AAV carrying a cell-type–specific promoter allows for brain cell type-specific transduction.[1–3] Moreover, changing the age of the mice for AAV injection allows for age-specific gene modification. There are multiple routes through which AAV can be administered to the brain. Direct injection into the brain parenchyma can cause high levels of expression with a relatively low dose, and expression in a limited area is achievable. However, it requires skillful techniques and an invasive surgical operation, especially when injecting it into a deep brain area. Moreover, a gradient of transgene expression centered around the injection needle tip is observed. Intravenous injection of mouse blood-brain barrier (BBB)–penetrating AAV[4–6] overcomes the disadvantages of direct parenchymal injection, and overall homogeneous and widespread transgene expression throughout the brain can be attained without a surgical procedure. However, a major disadvantage of intravenous injection is the requirement of a higher dose of AAV and the accompanying toxicity observed in the peripheral off-target tissues, such as the liver and muscle, that can sometimes make the interpretation of the observed phenotype difficult. Through both techniques (i.e., direct brain tissue and intravenous injections), the application of low-dose AAV results in transduction only in sparse cells, whereas high-dose AAV injections likely lead to leaky transgene expression in nontarget brain cells. Therefore, it is important to determine the optimal AAV dose to obtain highly efficient target cell type-specific transgene expression.

Received 13 June 2023; accepted 4 January 2024;
https://doi.org/10.1016/j.omtm.2024.101185.

Correspondence: Hirokazu Hirai, Department of Neurophysiology and Neural Repair, Gunma University Graduate School of Medicine, Maebashi, Gunma 371-8511, Japan.
E-mail: hirai@gunma-u.ac.jp

This is an open access article under the CC BY license (http://creativecommons.org/licenses/by/4.0/).

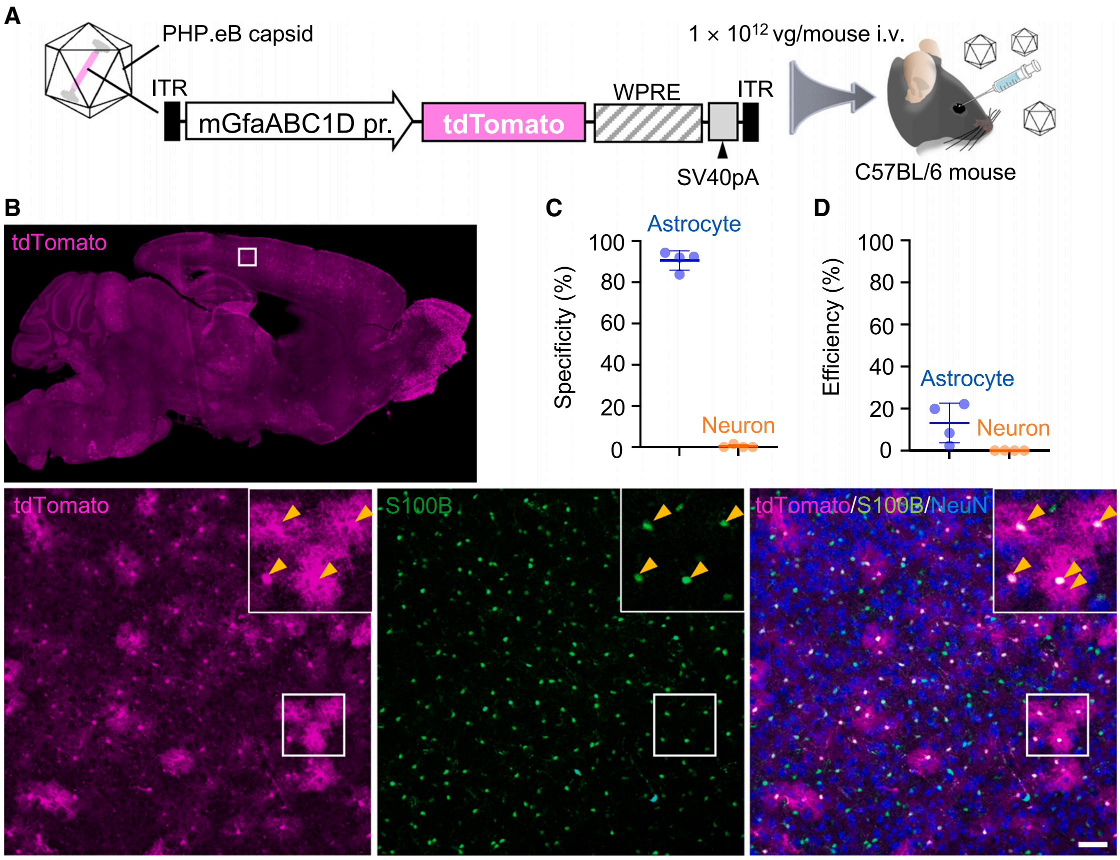

Figure 1. Astrocyte-specific transgene expression by AAV carrying the astrocyte-specific mGfaABC1D promoter

(A) Schema of AAV and experimental procedure. AAV vectors coated with PHP.eB, a BBB-penetrating capsid variant, which express tdTomato under control of the mouse GFAP (mGfaABC1D) promoter (100 μL, 1×10^{13} vg/mL), was injected into the C57BL/6 mouse through the orbital plexus. (B) Immunohistochemistry of mouse brain 3 weeks after AAV injection. Sagittal brain sections were immunostained for S100B, a marker of astrocytes, and NeuN, a marker of neurons. A square region in the tdTomato fluorescence image of sagittal brain section (top) was magnified (bottom), in which square regions were enlarged and presented at upper right corners. Arrowheads indicate S100B-labeled astrocytes. Scale bar, 50 μm. (C) Graph showing specificity for astrocyte transduction and that for neuronal transduction. Specificity for astrocyte (or neuron) transduction was calculated by the number of tdTomato and S100B (or NeuN) double-positive cells divided by the number of tdTomato^{+} cells. (D) Graph showing transduction efficiency for astrocytes and neurons. Transduction efficiency was calculated by dividing number of tdTomato and S100B (or NeuN) double-positive cells divided by number of S100B^{+} (or NeuN^{+}) cells. Data (average ± SD) were obtained from 4 mice, and the value from each mouse was plotted. ITR; inverted terminal repeat; SV40pA; simian virus 40 polyadenylation signal.

The glial fibrillary acidic protein (GFAP) promoter is often used to express transgenes specifically in astrocytes. Astrocyte-specific transgenic mice were initially produced using the Gfa2 promoter, a human genomic region extending 2.2 kb upstream of the *GFAP* RNA start site.[7] Subsequent transgenic mouse studies revealed that the truncated 681-bp promoter containing subregions A, B, C_1, and D (GfaABC1D promoter) worked specifically in astrocytes with 2-fold greater activity than the original 2.2-kb Gfa2 promoter.[8] Furthermore, AAV with the GfaABC1D promoter has been shown to induce astrocyte-specific transgene expression in rodents and rhesus macaques.[9–11] In this study, we aimed to achieve astrocyte-specific recombination in the floxed mouse brain by intravenously injecting BBB-penetrating AAV-PHP.eB (PHP.eB), which expresses Cre under the control of the GfaABC1D promoter in Ai14 reporter mice.

RESULTS

Astrocyte-specific transduction after systemic administration of PHP.eB with mouse GfaABC1D promoter

Previously, we demonstrated astrocyte-specific transduction by intravenous infusion of BBB-penetrating AAV-PHP.B with the astrocyte-specific mouse GfaABC1D (mGfaABC1D) promoter (Figure S1).[12] To confirm this, we produced PHP.eB[4] expressing tdTomato under the control of the mGfaABC1D promoter. The PHP.eB (1.0×10^{13} viral genome [vg]/mL, 100 μL) was intravenously infused to mature wild-type mice through the orbital plexus (Figure 1A). Three weeks after viral injection, cerebral sections were produced and double immunostained for S100B (an astrocyte marker) and NeuN (a neuron marker). tdTomato was not immunolabeled and assessed by intrinsic fluorescence throughout. Efficient transduction was observed throughout the brain (upper panel in Figure 1B). A magnified image

of the cerebral cortex showed numerous tdTomato^{+} cells, most of which were coimmunolabeled with S100B (lower panels in Figure 1B). Quantitative analysis showed that over 90% of tdTomato^{+} cells were astrocytes immunolabeled for S100B (90.6% ± 4.1%, n = 4 mice), with only a minor fraction of tdTomato^{+} cells being NeuN^{+} neurons (0.4% ± 0.7%, n = 4 mice) (Figure 1C), indicating astrocyte-specific transduction by PHP.eB with the mGfaABC1D promoter, which is consistent with a previous report.[12]

Next, we calculated the transduction efficiency of astrocytes in the cerebral cortex, which is the ratio of the number of tdTomato^{+} and S100B^{+} cells to the total number of S100B^{+} astrocytes in the cerebral cortex. The transduction efficiency of neurons in the same region was calculated by dividing the number of tdTomato^{+} and NeuN^{+} cells by the total number of NeuN^{+} neurons. The results showed that approximately 13% of astrocytes and almost none of neurons were transduced (Figure 1D).

Absence of astrocyte-specific transduction in Ai14 mice by intravenous infusion of PHP.eB expressing Cre under control of mGfaABC1D promoter

To clarify whether the astrocyte-targeted transduction by PHP.eB with the mGfaABC1D promoter can be applied to floxed mice, we intravenously injected the same dose (1.0×10^{12} vg/mouse) of PHP.eB expressing Cre recombinase under the control of the mGfaABC1D promoter in mature Cre-reporter Ai14 mice (Figure 2A). Ai14 mice have a loxP-flanked STOP cassette in the Gt(ROSA)26Sor locus, which prevents the transcription of a CAG promoter-driven tdTomato. Ai14 mice trigger tdTomato expression following Cre-mediated removal of the STOP cassette. Thus, we expected astrocyte-specific tdTomato expression in Ai14 mice systemically treated with PHP.eB expressing Cre under the control of the mGfaABC1D promoter. To validate this, sagittal brain sections of the injected Ai14 mice were produced 3 weeks after AAV treatment, followed by immunolabeling for S100B and NeuN. However, we could not distinguish between the cell types in the cerebral cortex because of the overwhelming expression of tdTomato without gaps between the cells (Figures 2B and S2A). Therefore, we reduced the injection dose to 0.2, 0.04, 0.02, and 0.01 times the original dose (1.0×10^{12} vg/mouse).

We identified individual cell outlines in the cerebral cortex of mice that received AAV vectors at $<4 \times 10^{10}$ vg/mouse (Figures 2C and S2A). Quantitative analysis in mice treated with AAV vectors at 4×10^{10} vg/mouse revealed that only 13% of tdTomato-expressing cells were S100B^{+} astrocytes and approximately 40% of tdTomato-expressing cells were NeuN^{+} neurons (Figure 2D). Immunohistochemistry using anti-CD31 and anti-Olig2 antibodies revealed that the remaining tdTomato^{+} cells were primarily brain microvascular endothelial cells (Figure S2B). The specificity for astrocyte transduction (∼10%) and that for neuron transduction (∼40%) was almost stable even with a further decrease in the viral doses, although the transduction efficiency of astrocytes decreased along with lowering the viral doses injected (Figure 2D).

Significant increase in astrocyte recombination by switching AAV capsid from PHP.eB to AAV-F

Previous studies have shown that PHP.eB and PHP.B were neurotropic,[4,13] whereas AAV-F, a different BBB-penetrating capsid variant, has a significantly higher tropism for astrocytes than PHP.B.[6] Therefore, switching from the PHP.eB capsid to the AAV-F may upregulate astrocyte recombination. To test this, we compared the transduction profile of AAV-F with that of PHP.eB, using a nonselective chicken β-actin hybrid intron (CBh) promoter. AAV-F or PHP.eB expressing EGFP under the control of the CBh promoter (2.0×10^{11} vg/mouse) were intravenously injected into C57BL/6 mice, and the brains were examined 3 weeks after the injection (Figure S3A).

GFP fluorescence intensity in the whole brain treated with PHP.eB was significantly higher than that of AAV-F (Figure S3B). Subsequently, we produced sagittal brain sections immunostained for S100B, NeuN, and Olig2, and examined the ratios of transduced astrocytes, neurons, and oligodendrocytes to the total number of transduced cells in the primary motor cortex. We found a significantly higher ratio of GFP-expressing astrocytes to total transduced cells in brains treated with AAV-F (51.7% ± 6.2%, n = 4 mice), compared with PHP.eB (24.2% ± 5.1%) ($p < 0.01$) (Figures S3C and S3D). Consistently, the ratio of transduced neurons to total transduced cells was significantly lower in AAV-F-treated brains than in PHP.eB-treated brains (Figures S3C and S3E). Almost no transduced oligodendrocytes were observed in either group (Figures S3C and S3F). These results suggested that AAV-F is more suitable for astrocyte transduction.

We then prepared AAV-F or PHP.eB, both of which express Cre under the control of the mGfaABC1D promoter. Ai14 mice received intravenous infusion of AAV-F or PHP.eB (4.0×10^{10} vg/mouse) and were examined by immunohistochemistry 3 weeks after injection (Figure 3A). As expected, replacement of the capsid from PHP.eB to AAV-F significantly increased the ratio of transduced astrocytes to total transduced cells from 9.3% ± 3.5% to 33.6% ± 5.6% (n = 4 mice, p = 0.0007) without affecting efficiency for astrocyte transduction (Figures 3B and 3C). Consistent with the increase in astrocyte transduction, utilization of the AAV-F capsid significantly decreased the ratio of neuronal transduction to total transduced cells from 49.7% ± 5.2% to 19.6% ± 1.8% (n = 4 mice, $p < 0.0001$), and efficiency of neuronal transduction from 17.3% ± 7.0% to 3.1% ± 1.2% (n = 4 mice, p = 0.0134) (Figure 3C).

Highly specific astrocyte transduction by combination of a codon-optimized flippase (FlpO)-destabilized Cre (dCre) system with AAV-F

The mGfaABC1D promoter works in an astrocyte-specific manner, but likely possesses subtle promoter activity in nonastrocytic cells. Thus, we hypothesized that Cre, which is expressed and accumulates in nonastrocytic cells, causes recombination and subsequent tdTomato expression in a broad range of cell types, including neurons, in the Ai14 mouse brain. In this case, strictly limiting Cre expression to astrocytes may inhibit nonastrocytic transduction.

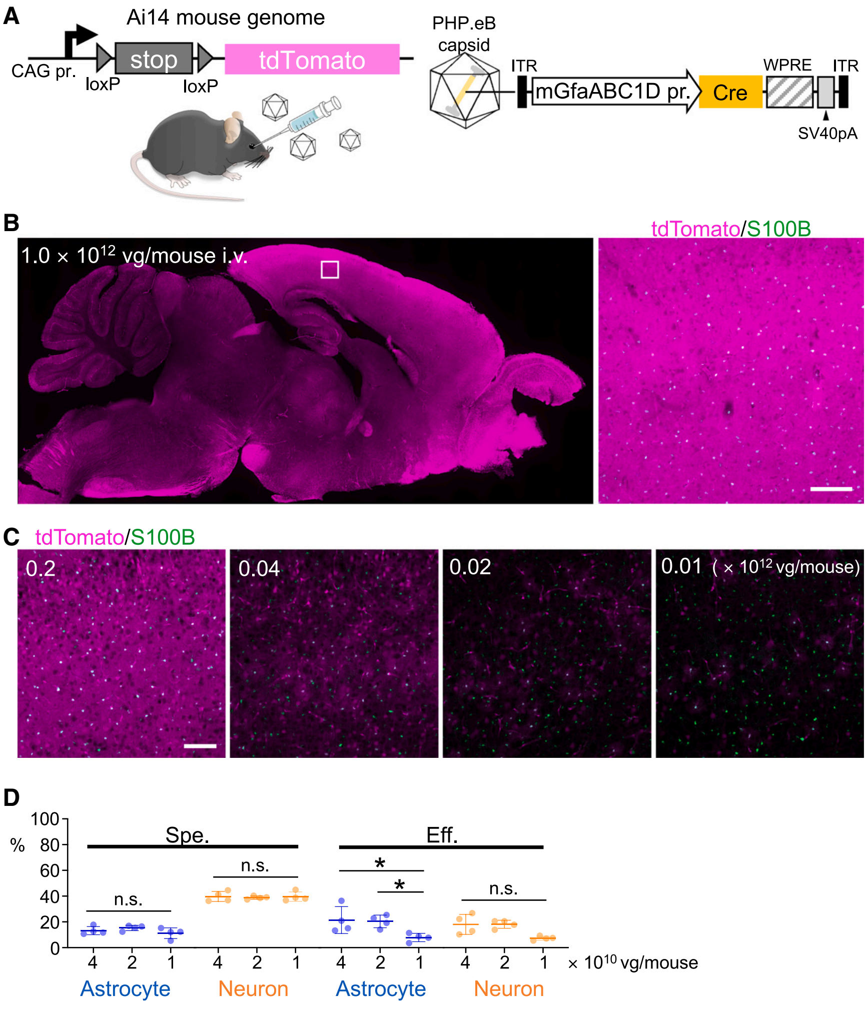

Figure 2. Nonspecific cellular transduction in floxed mouse brain by intravenously administered PHP.eB expressing Cre by mGfaABC1D promoter

(A) Schema showing experimental procedure. An Ai14 reporter mouse, which has the CAG promoter, bilaterally loxP-flanked STOP cassette, and cDNA of tdTomato, a red fluorescent protein, received intravenous injection of PHP.eB expressing Cre by mGfaABC1D promoter. (B) Immunohistochemistry of mouse brain 3 weeks after AAV injection (100 μL, 1 × 10^{13} vg/mL). Left, sagittal brain section immunostained for S100B. Right, magnification of a square region at left; tdTomato expression was too strong to distinguish individual cells. (C) Immunohistochemistry of cerebral cortex from mice that were similarly treated with (B), except for injection dose of AAV. Those mice received lower doses of AAV ranging from one-fifth to one-one hundredth. (D) Graphs showing specificity (Spe., left) and efficiency (Eff., right) for transduction of astrocytes and neurons. Data (average ±SD) were obtained from 4 mice, and the value from each mouse was plotted. n.s., not significant and *p < 0.05 by 1-way ANOVA with Bonferroni's post hoc test. Scale bars, 100 μm.

To validate our hypothesis, we used a FlpO-flippase recognition target (FRT) system, which is analogous to but less efficient than the Cre-*loxP* system. In addition, we generated dCre by fusing Cre recombinase with a PEST sequence, a segment rich in proline, glutamate, serine, and threonine from murine ornithine decarboxylase, to facilitate degradation.[14,15] In this strategy, Ai14 mice received an intravenous infusion of a mixture of two different PHP.eB capsid vectors carrying the mGfaABC1D promoter—one expressing FlpO and the other expressing dCre in response to FlpO-mediated recombination (Figure 4A). The mGfaABC1D promoter likely drives the expression of a small amount of FlpO in nonastrocytic cells; however, FlpO causes recombination 10 times less efficiently than Cre.[16] Eventually, a smaller population of nonastrocytic cells is thought to express dCre. Moreover, the expressed dCre was rapidly degraded because of the appended PEST sequence.[17]

A mixture of PHP.eB vectors expressing FlpO by the mGfaABC1D promoter and PHP.eB expressing dCre by the mGfaABC1D promoter in the presence of FlpO (5.0 × 10^{11} vg/mouse) was intravenously administered to Ai14 mice (Figure 4A). Three weeks after the injection, sagittal sections of the brain were obtained and immunostained for S100B and NeuN. Immunohistochemistry of the cerebral cortex showed numerous tdTomato-expressing cells, in which approximately 50% were S100B-expressing astrocytes (49.9% ± 10.9%, n = 4 mice) and 50% were NeuN-labeled neurons (47.1% ± 10.0%, n = 4 mice) (Figures 4B and 4C). The recombination efficiencies of astrocytes and neurons were 43.3% ± 6.8% and 14.7% ± 4.3%, respectively (Figure 4D). These results suggest that the FlpO-dCre expression system with two different AAV vectors significantly increased the specificity and efficiency of astrocyte transduction in floxed mice compared with a single AAV expressing Cre.

Based on these results, we combined the astrocyte-tropic AAV-F capsid with the FlpO-dCre system to target astrocytes. The mixture of AAV-F vectors expressing FlpO by the mGfaABC1D promoter and AAV-F expressing dCre by the mGfaABC1D promoter in the presence of FlpO (5.0 × 10^{11} vg/mouse, respectively) was systemically administered to Ai14 mice (Figure 5A). Immunohistochemistry of treated brains revealed numerous tdTomato$^+$ cells, most of which were immunolabeled for S100B (Figure 5B). Quantitative analysis showed that >90% of tdTomato$^+$ cells were S100B-expressing astrocytes (92.6% ± 1.4%, n = 4 mice), with only 5.0% ± 0.4% of tdTomato$^+$ cells being NeuN$^+$ neurons (Figure 5C). As for the transduction efficiency, >40% of astrocytes and <1% of neurons expressed tdTomato (42.8% ± 9.7% and 0.7% ± 0.1%, respectively, n = 4 mice) (Figure 5D).

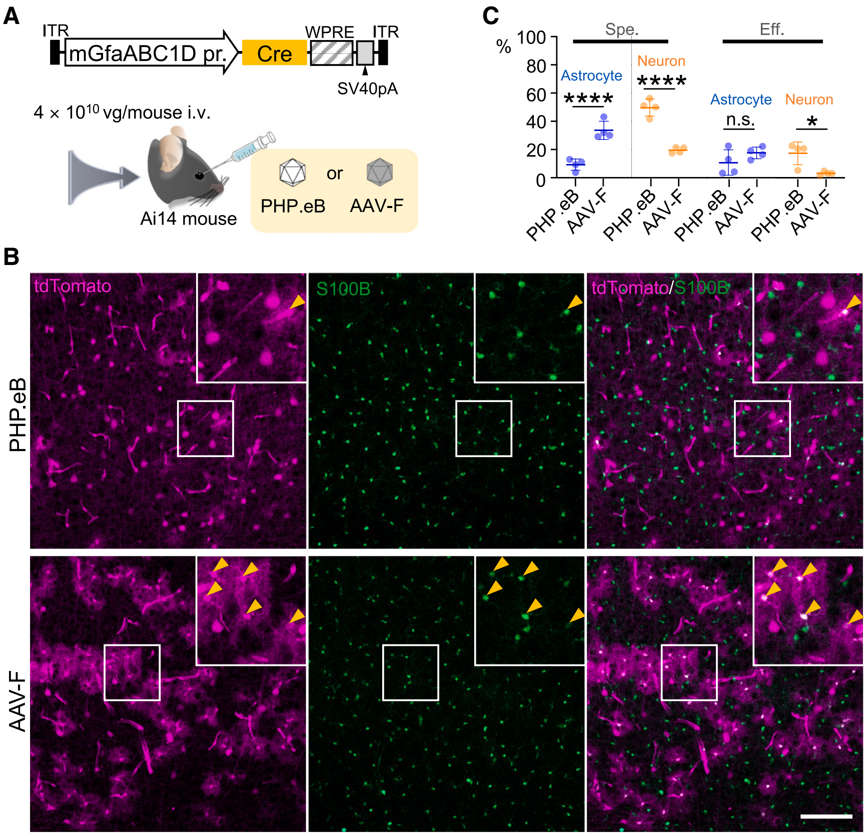

Figure 3. Significant upregulation of astrocyte transduction by shifting AAV capsid from PHP.eB to AAV-F

(A) Schema showing experimental procedure. Ai14 reporter mouse received intravenous injection of PHP.eB or AAV-F expressing Cre by mGfaABC1D promoter (100 μL, 4×10^{11} vg/mL). (B) Immunohistochemistry of mouse brain 3 weeks after AAV injection. Astrocytes were immunolabeled for S100B. Fluorescence images of primary motor cortex from PHP.eB-treated (top) and AAV-F-treated (bottom) mice were presented, in which square regions were enlarged to present at respective upper right corners. Arrowheads indicate S100B-immunolabeled astrocytes. Scale bar, 100 μm. (C) Graph showing specificity (Spe.) and efficiency (Eff.) for astrocyte transduction (left) and that for neuronal transduction (right) in mice treated with PHP.eB or AAV-F vectors. Data (average ± SD) were obtained from 4 mice, and the value from each mouse was plotted. n.s., not significant; *$p < 0.05$; and ****$p < 0.0001$ by unpaired Student's t test.

Because enhanced astrocyte specificity may be attained using dCre alone (without FlpO), the AAV-F expressing dCre with the mGfaABC1D promoter (4.0×10^{10} vg/mouse intravenously) were injected into the Ai14 mice (Figure S4). However, this did not increase but rather decreased the specificity and efficiency of astrocyte transduction (Table S1), suggesting that using dCre in combination with the mGfaABC1D promoter did not increase the specificity or efficiency.

Neuron-targeted recombination in floxed mice by FlpO-dCre method

Next, we examined whether the FlpO-dCre system combined with a capsid tropic for a target cell population could be used for neuronal targeting. We first tested unmatched capsids, that is, astrocyte-tropic AAV-F-expressing Cre, under the control of the neuron-specific enolase (NSE) promoter.[3,12] AAV-F vectors expressing Cre under the control of the NSE promoter were injected intravenously into Ai75G mice expressing tdTomato fused with a nuclear localization signal (NLS) sequence in the presence of Cre (Figure 6A). Immunohistochemistry 3 weeks after the AAV injection showed that among tdTomato-expressing cells, 68% were NeuN-labeled neurons and >20% of cells were S100B-labeled astrocytes, despite using the neuron-specific NSE promoter (upper panels in Figures 6B and 6C). Switching the capsid from AAV-F to neuron-tropic PHP.eB (expressing Cre by the NSE promoter) significantly increased the specificity for neuronal transduction from 68% to 78% and efficiency for neuronal transduction from 69% to 94% (center panels in Figures 6B and 6C).

Finally, we tested the most favorable combination, neurotropic PHP.eB with the FlpO-dCre system. Systemic infusion of the mixture of PHP.eB expressing FlpO and PHP.eB expressing dCre in the presence of FlpO to Ai75G mice (Figure 6A) attained highly specific and efficient neuronal transduction (88.1% ± 4.5% and 83.1% ± 14.9%, respectively), with complete elimination of astrocyte transduction (lower panels in Figures 6B and 6C). These results suggest that the FlpO-dCre system combined with an AAV capsid tropic in a target cell population can be applied for the transduction of cell types other than astrocytes.

Combination of FlpO-dCre with a capsid tropic to a target cell type also works in brain parenchymal injection

Next, we examined whether FlpO-dCre in combination with a cell-type–specific capsid also worked in brain parenchymal injections. In the direct cortical injection, we used the AAV5 capsid because it was shown to be significantly tropic to astrocytes.[18] AAV5 expressing Cre under the control of the mGfaABC1D promoter (5.0×10^8 vg/mouse) was injected into the primary cortex of Ai14 mice (Figure 7A). Immunohistochemistry at 3 weeks postviral injection showed numerous tdTomato-expressing but S100B$^-$ (i.e., nonastrocytic) cells (Figure 7B), which was analogous to the result of the systemic injection of AAV-F-mGfaABC1D promoter-Cre (Figure 3).

Next, we injected a mixture of AAV5 expressing FlpO under the control of the mGfaABC1D promoter and AAV5 expressing dCre under the control of the mGfaABC1D promoter in an FlpO-dependent manner (2.5×10^8 vg/mouse) (Figure 7C). Immunohistochemistry

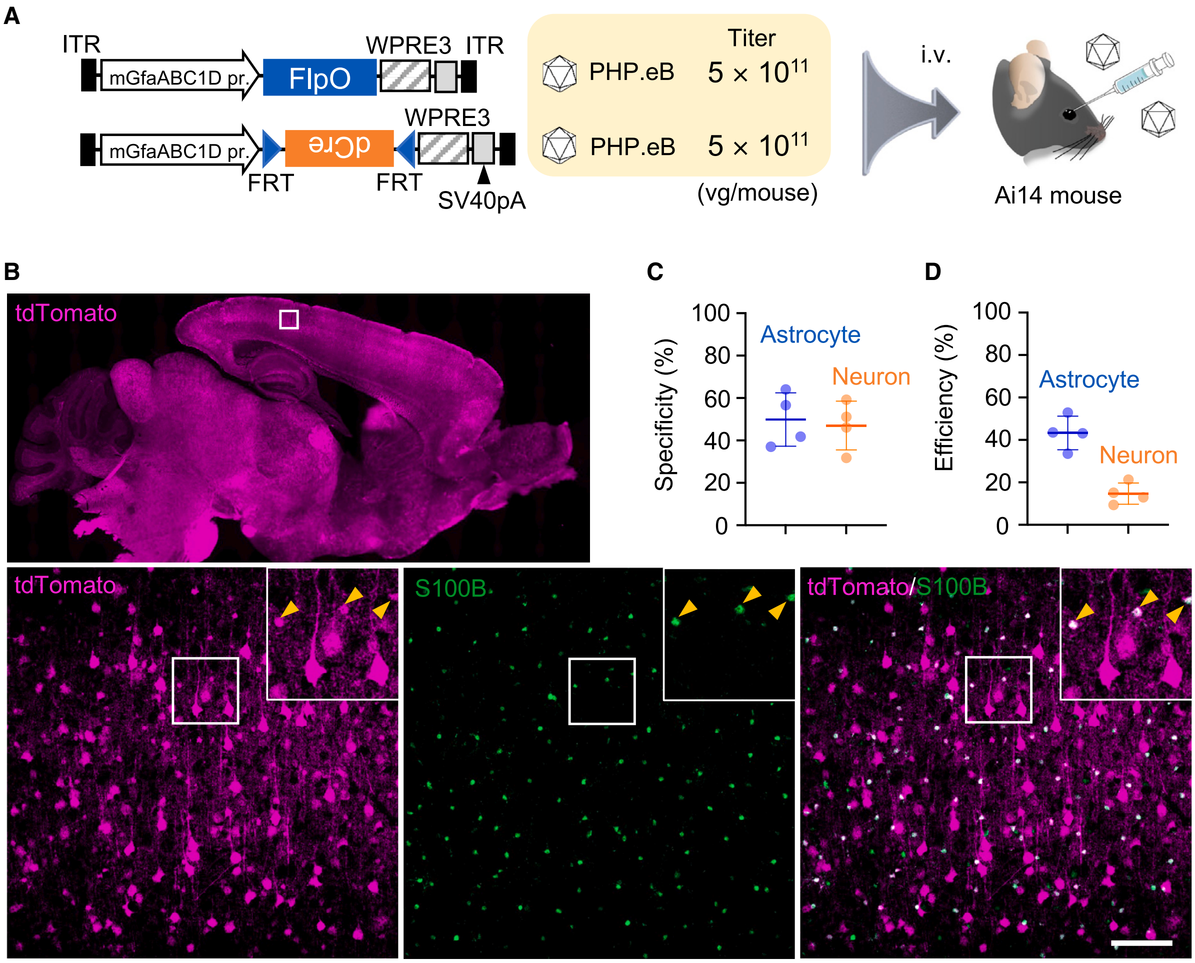

Figure 4. Increase in specificity and efficiency for astrocyte transduction by FlpO-dCre system

(A) Schema showing structure of AAV vectors and experimental procedure. Two AAV vectors were used: one carrying mGfaABC1D promoter-driven mouse codon-optimized flippase recombinase (FlpO) and another carrying mGfaABC1D promoter-driven short FRT site-flanked, inverted dCre. Thus, dCre expresses in the presence of FlpO in cells infected with both AAV vectors. PHP.eB-coated AAV mixture (5×10^{11} vg/mouse, respectively) was injected into Ai14 mice through the orbital plexus. (B) Immunohistochemistry of mouse brain 3 weeks after AAV injection. A square region in tdTomato fluorescence image of sagittal brain section (top) was magnified (bottom), in which square regions were enlarged and presented at upper right corners. Arrowheads indicate S100B-labeled astrocytes. Scale bar, 100 μm. (C and D) Graph showing specificity (C) and efficiency (D) for astrocyte and neuronal transduction. Data (average ± SD) were obtained from 4 mice, and the value from each mouse was plotted.

performed 3 weeks after injection showed numerous tdTomato$^+$ and S100B$^+$ astrocytes (Figure 7D). Although astrocyte specificity by AAV5- mGfaABC1D promoter-Cre was as low as 34.0% ± 8.5%, a mixture of two AAV5 vectors with the FlpO-dCre system significantly increased the astrocyte specificity to 73.6% ± 1.1% (Figure 7E). These results suggest that our FlpO-dCre system, in combination with a capsid tropic to a target cell type, is also available for direct cortical injection into floxed mice.

Transduction profiles of astrocytes present in the brain region apart from the cerebral cortex by AAV-F/FlpO-dCre system

The possibility that the AAV-F/FlpO-dCre system causes astrocyte-targeted recombination in other brain regions was assessed. Ai14 mice were injected with a mixture of AAV-F vectors expressing FlpO and dCre, as depicted in Figure 5A. The results are summarized in Figure S5 and Table S2. With respect to astrocyte recombination, the thalamus and hippocampus showed over 80% specificity, followed by the olfactory bulb (62.0% ± 14.1%) and the caudate putamen (37.3% ± 21.4%) (Table S2). Moreover, with respect to the recombination efficiency, the olfactory bulb had the highest efficiency (48.7% ± 16.3%) and the thalamus had the second highest efficiency (48.7% ± 16.3%), followed by the hippocampus (22.3% ± 5.7%) and the caudate putamen (8.7% ± 5.9%). However, the tdTomato$^+$ cells were not detected in the corpus callosum.

DISCUSSION

In the present study, we found that Cre expression by systemic or local administration of AAV with an astrocyte-specific mGfaABC1D

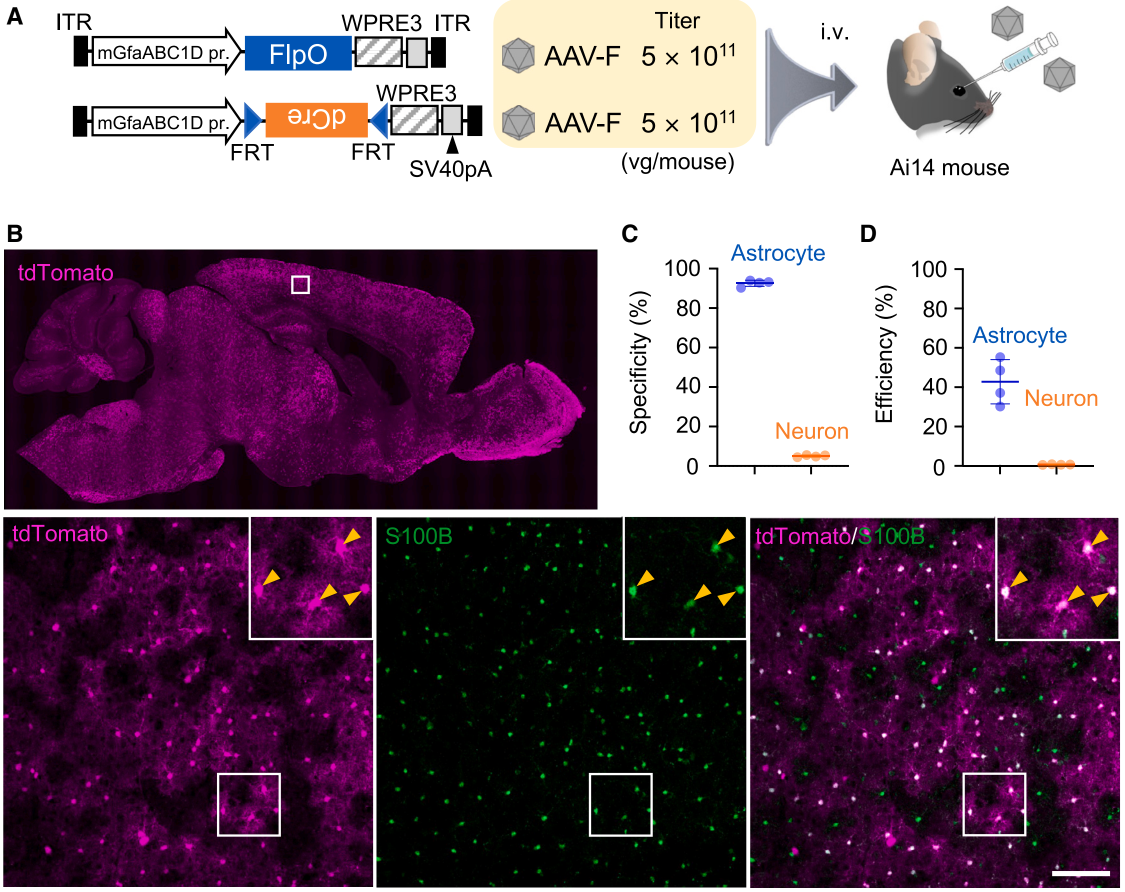

Figure 5. Astrocyte-specific transduction by intravenous infusion of 2 AAV-F mixtures expressing dCre in the presence of FlpO

(A) Schema showing experimental procedure. AAV genomes, which were the same as those in Figure 4, were coated with BBB-penetrating AAV-F capsid, instead of PHP.eB. AAV-F mixture (5×10^{11} vg/mouse, respectively) was injected into Ai14 mice through the orbital plexus. (B) Immunohistochemistry of mouse brain 3 weeks after the AAV injection. A square region in the tdTomato fluorescence image of sagittal brain section (top) was magnified (bottom), in which square regions were enlarged and presented at upper right corners. Arrowheads indicate S100B-labeled astrocytes. Scale bar, 100 μm. (C and D) Graph showing specificity (C) and efficiency (D) for astrocyte and neuronal transduction. Data (average ± SD) were obtained from 4 mice, and the value from each mouse was plotted.

promoter caused nonspecific recombination in the floxed Ai14 mouse brain, including both neurons and astrocytes. Moreover, neurons were the predominant cell type despite the use of an astrocyte-specific promoter. We hypothesized that the paradoxical outcome was caused by mGfaABC1D promoter-driven, slight but continuous expression and accumulation of Cre in nonastrocytic cells, leading to nonspecific recombination, whereas recombination in astrocytes was inhibited by the excess expression of Cre, likely due to the formation of Cre-DNA aggregates.[19] Thus, it would be necessary to suppress leaky Cre expression in nontarget cells while maintaining Cre at the optimum dose in target cells.

To suppress the overall expression of Cre in both astrocytes and non-astrocytic cells, we decreased the injection dose of AAV vectors. Serial lowering of the injection dose significantly decreased the transduction efficiency of astrocytes; however, it did not influence the specificity of astrocyte transduction (Figure 2D). After several trials, we successfully restricted the recombination specifically to astrocytes by using the FlpO-dCre system in combination with an astrocyte-tropic AAV-F capsid. Likewise, neuron-specific recombination was attained using the NSE promoter-driven FlpO-dCre expression system together with a neuron-tropic PHP.eB capsid. Furthermore, this strategy was shown to be available not only for systemic administration but also for direct cortical injection.

Our FlpO-dCre system with a target cell-tropic capsid succeeded in achieving the above conditions using at least three different means. The first is the use of a target cell-tropic capsid, which decreases the number of AAV copies to infect nontarget cells. The second is the use of FlpO, which has only one-tenth the recombinase activity of Cre.[16] The third mechanism is division of one AAV into two AAVs, which enables the dual use of a cell-type–specific promoter and thereby synergistically limits the expression of dCre in nontarget cells. By contrast, the use of dCre may not contribute to the system

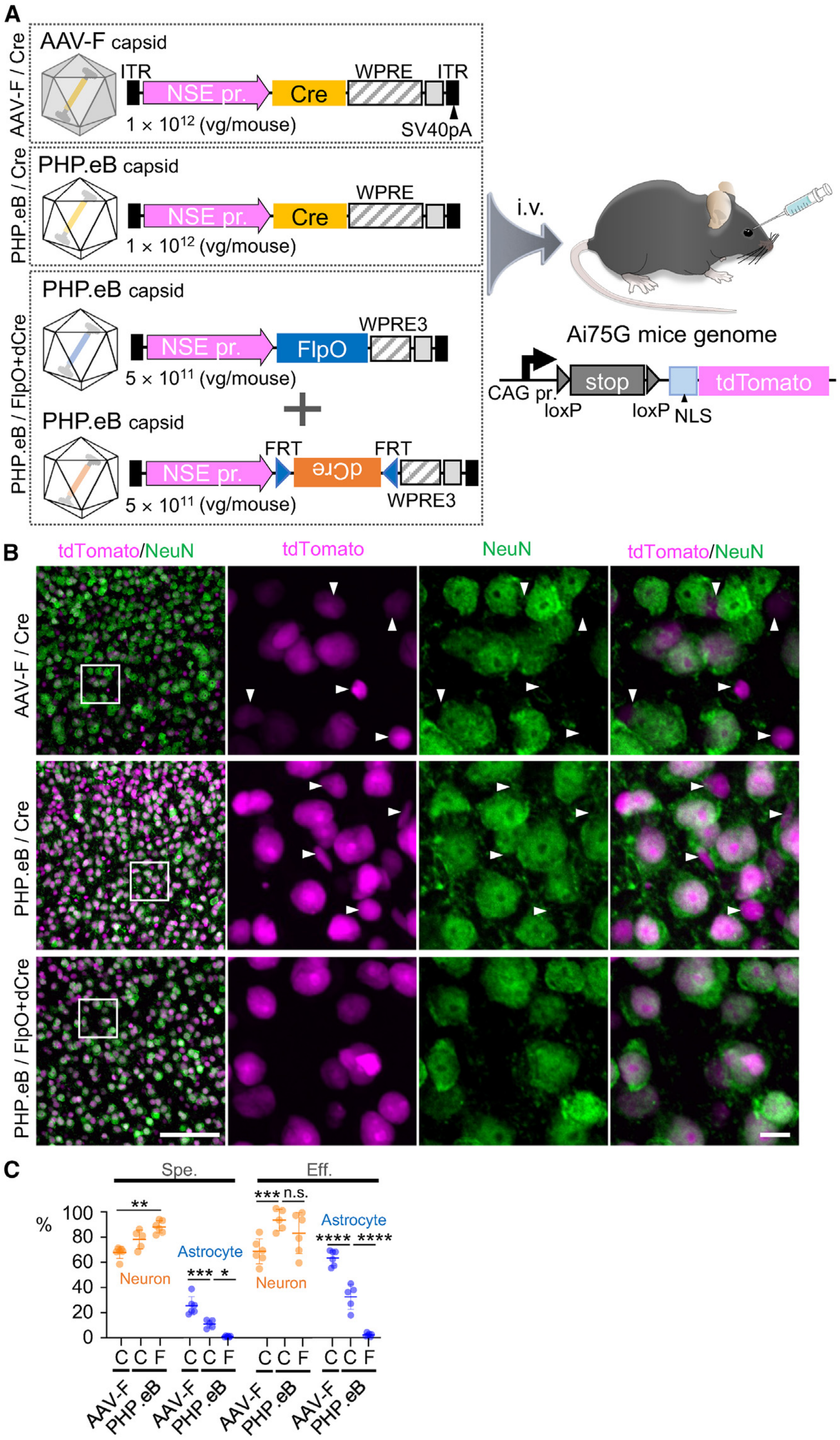

Figure 6. Neuron-specific transduction in floxed mice by FlpO-dCre system in combination with a neurotropic PHP.eB capsid

(A) Schema showing experimental procedure. Ai75G mice, which express NLS-attached tdTomato in the presence of Cre, received intravenous injection of astrocyte-tropic AAV-F or neuron-tropic PHP.eB capsid vectors expressing Cre under control of NSE promoter, as controls. To prove the efficacy of our FlpO-dCre system, Ai75G mice similarly received a mixture of 2 NSE promoter-driven PHP.eB vectors: one expressing FlpO and another expressing dCre in the presence of FlpO. (B) Immunohistochemistry of mouse brain 3 weeks after AAV injection. Far left, fluorescence images of tdTomato (magenta) and NeuN (green) from primary motor cortex, in which square regions were enlarged and arranged at right. Arrowheads indicate tdTomato^{+} and NeuN^{-} nonneuronal cells. Scale bars in bottom left and bottom right, 100 and 10 μm, respectively. (C) Graph showing specificity (Spe.) and efficiency (Eff.) for the transduction of neurons and astrocytes. “C” and “F” below horizontal axis indicate “Cre” and “FlpO-dCre,” respectively. Data (average ± SD) were obtained from 4 mice, and the value from each mouse was plotted. n.s., not significant; $^{*}p < 0.05$; $^{**}p < 0.01$; $^{***}p < 0.001$; and $^{****}p < 0.0001$ by 1-way ANOVA with Bonferroni’s post hoc test.

outcome, because switching from Cre to dCre in AAVs with the mGfaABC1D promoter did not increase the specificity or efficiency (Table S1). In this experiment, we assessed the treated mice 3 weeks after the AAV injection. The amount of Cre in the infected cells increases over time; thus, the use of dCre may become effective after 3 weeks of AAV injection.

A previous study by Gao et al. reported similar leaky neuronal expression following subretinal injection of the AAV-GfaABC1D promoter-Cre-WPRE (woodchuck hepatitis virus post-transcriptional regulatory element) in Ai9 floxed mice.[20] To suppress leaky Cre expression in nontarget retinal ganglion cells, the WPRE sequence was removed from the AAV genome (AAV-GfaABC1D promoter-Cre-ΔWPRE) and the retinal ganglion cell transduction was eliminated without compromising the specificity and efficiency of the target Müller glia transduction. Thus, AAV-GfaABC1D promoter-Cre-ΔWPRE may also achieve astrocyte-specific recombination in the floxed mouse brain.

Our method can be applied to study unknown gene functions by inducing recombination in floxed mice in a cell-type– and age-specific manner. Systemic administration of BBB-penetrating AAV causes whole-brain transduction, whereas local brain parenchymal injection enables the recombination of a limited brain region. Cell types that undergo recombination can be changed easily by switching the cell-type–specific promoters and the capsids tropic to target cell types, indicating that this method

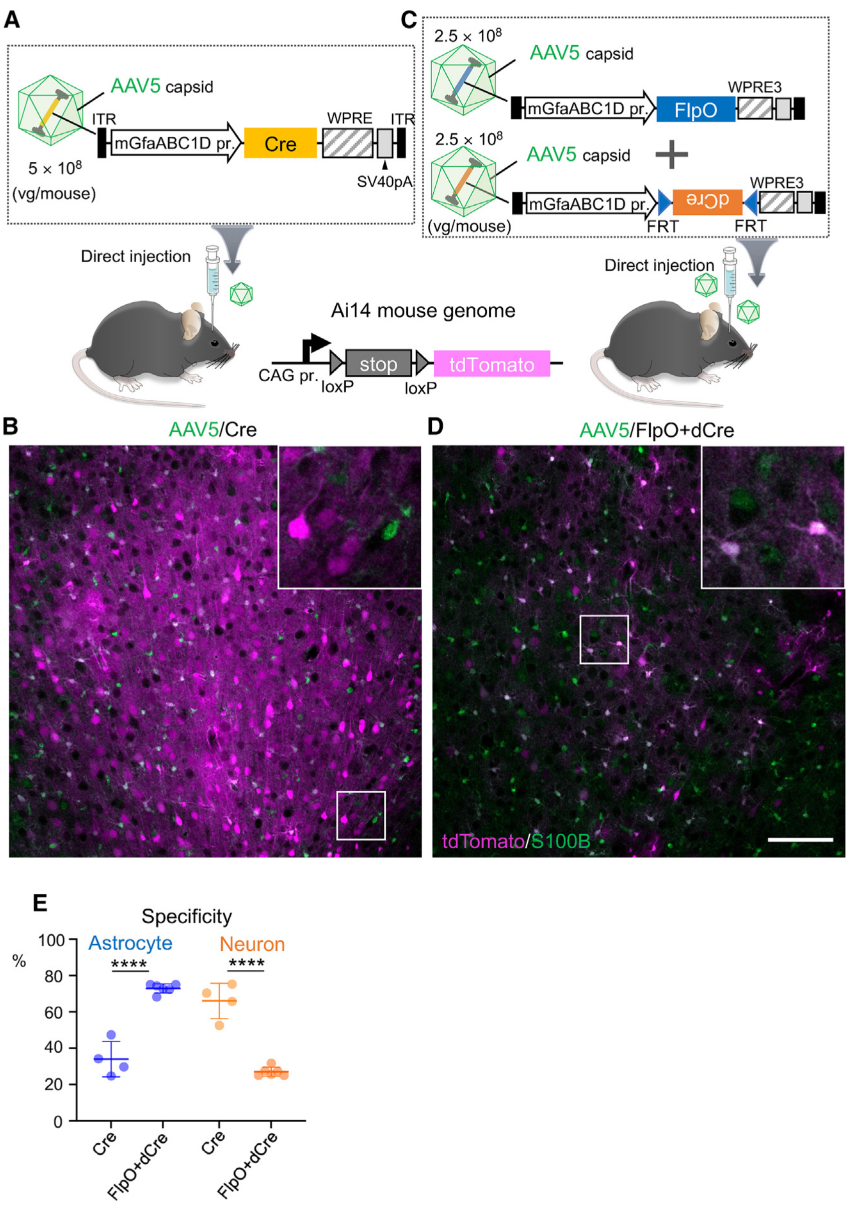

Figure 7. FlpO-dCre system in combination with a target cell–tropic capsid is also available for direct cortical injection

(A and C) Schema showing experimental procedure. Ai14 mice received intravenous injection of astrocyte-tropic AAV5 capsid vectors expressing Cre under control of mGfaABC1D promoter (A) or mixture of mGfaABC1D promoter-driven AAV5 vectors: one expressing FlpO and another expressing dCre in the presence of FlpO (C). (B and D) Immunohistochemistry of mouse brain 3 weeks after AAV injection. Fluorescence images show primary motor cortex from mice treated with AAV5 expressing Cre (B) and those treated with AAV5 mixture expressing FlpO and dCre (D). Square regions were enlarged and are shown in upper right. Note that there are numerous tdTomato-expressing and $S100B^{-}$ nonastrocytic cells (B), whereas most tdTomato-expressing cells were immunolabeled also for S100B (D). Scale bar, 100 μm. (E) Graph showing specificity for astrocyte (left) and that for neuron (right) in Ai14 mice treated with AAV5 as depicted in (A) and (C). Data (average ± SD) were obtained from 3–4 mice, and the value obtained from each mouse was plotted. ****$p < 0.0001$ by unpaired Student's t test.

can be used to screen gene functions in different cell types with substantial savings in time and effort.

As shown in the present study, a BBB-penetrating capsid variant with high neuron infectivity (PHP.eB) and that with high astrocyte infectivity (AAV-F) are available. However, such variants with high infectivity in other cell types have not yet been well established. Moreover, the cellular tropism of PHP.eB and AAV-F likely differed depending on the region of the brain, leading to nontarget cell recombination in regions other than the cerebral cortex. Notably, large regional differences in the specificity and efficiency of astrocyte transduction were observed in our FlpO-dCre method combined with AAV-F (Figure S5; Table S2). Therefore, if current AAV doses (5.0×10^{11} vg/mouse, respectively) are used, then our method is useful in the cortex, hippocampus, and thalamus, but may be less reliable in other brain regions. To target other brain regions, the dose of AAV should be adjusted and different capsids should be tested to achieve higher specificity and efficiency.

Our present method attained highly specific target cell recombination in the cerebral cortex at $\sim\geq$90%, almost comparable to a conventional method using cell-type–specific CreER-driver mice (~87%).[21] Regarding the transduction efficiency, our method using PHP.eB and the NSE promoter allowed >83%, whereas the transduction efficiency for astrocytes using AAV-F and the mGfaABC1D promoter remained as low as 43%. Meanwhile, ~75% of astrocytes were transduced by the conventional method using a mouse line expressing CreER by the human GFAP promoter.[21] The relatively low transduction efficiency of astrocytes may be due to the need for two different AAV vector infections in one cell for recombination. Systemic injection of a single AAV-F vector at 5.0×10^{11} vg/mouse transduces 60%–70% astrocytes, resulting in <50% cotransduction when two different AAVs are used. Accordingly, to gain >50% cotransduction efficiency, injection doses should be increased. However, this could increase leaky Cre expression in nontarget cells, resulting in reduced specificity of astrocyte transduction.

The method described in this study requires the administration of an AAV dose ~10 times higher to attain cotransduction for the

expression of two recombinases, which increases the risk of immune activation and endoplasmic reticulum stress. Therefore, if a single Cre-expressing AAV vector, such as an AAV vector lacking WPRE,[20] works in the brain, it could reduce the injection dose and potential neurotoxicity, and thus could be a better alternative than the current two AAV vector system.

MATERIALS AND METHODS

AAV vector preparation

We used BBB-penetrating AAV-PHP.eB (PHP.eB)[4] and AAV-F[6] capsid variants. The pAAV expression plasmid comprises an astrocyte-specific mGfaABC1D, neuron-specific NSE promoter, ubiquitous CBh promoter, WPRE or WPRE3,[22] and a simian virus 40 (SV40) polyadenylation signal sequence.[12] The mGfaABC1D promoter was constructed according to a previous report.[23] The transgenes incorporated were *tdTomato*, *GFP*, *Cre*, *FlpO*, and flp-dependent *dCre*. They were then inserted into the AgeI/NotI-digested sites of pAAV vectors. The packaging plasmid for PHP.eB was constructed by replacing the wild-type fragment between the BsiWI and PmeI sites of pAAV2/9 (kindly provided by Dr. J. Wilson, University of Pennsylvania) with a mutant capsid gene fragment. AAV-F was constructed by replacing the wild-type fragment between HindIII and EcoRV of pAAV2/9 with a humanized mutant capsid gene fragment. Two fragments with the following primers containing the insertion sequence for FVVGQSY of AAV-F based on AAV2/9 were amplified by PCR using KOD One PCR Master Mix (Toyobo, Osaka, Japan) and inserted into a plasmid cut with HindIII and EcoRV using the In-Fusion HD Cloning Kit (Takara Bio, Shiga, Japan): F1-forward: 5′-TCAGACGCGGAAGCTTCGATCAAC-3′, F1-reverse: 5′-GTAGCTCTGGCCCACGACGAATTGGGCACTCT GGTGGTTTGTGG-3′, F2-forward: 5′- GTGGGCCAGAGCTACGC ACAGGCGCAGACCGGCTGGG-3′, F2-reverse: 5′- CAGTGTGAT GGATATCTGCAGAATTC-3′. Recombinant single-stranded PHP.eB, AAV-F, and AAV5 vectors were produced using an ultracentrifugation method as described in a previous paper.[24] Briefly, HEK293T cells (HCL4517; Thermo Fisher Scientific, Waltham, MA), which were cultured in DMEM (D5796-500ML, Sigma-Aldrich, St. Louis, MO) supplemented with 8% fetal bovine serum (Sigma-Aldrich), were transfected with three plasmids: the expression plasmid (pAAV/mGfaABC1D-tdTomato-WPRE-SV40pA, pAAV/mGfaABC1D-Cre-WPRE-SV40pA, pAAV/mGfaABC1D-FlpO-WPRE3-SV40pA, pAAV/mGfaABC1D-frt-inverted dCre-frt-WPRE3-SV40pA, pAAV/NSE-Cre-WPRE-SV40pA, pAAV/NSE-FlpO-WPRE3-SV40pA, pAAV/NSE-frt-inverted dCre-frt-WPRE3-SV40pA, or pAAV/CBh-GFP-WPRE-SV40pA), the pHelper (Agilent Technologies, Santa Clara, CA), and the packaging plasmid (pAAV-PHP.eB, pAAV-F, or pAAV2/5). Viral particles were harvested from the culture medium 6 days after transfection and concentrated by precipitation with 8% polyethylene glycol 8000 (P5413; Sigma-Aldrich) and 500 mM sodium chloride. The precipitated particles were resuspended in Dulbecco's PBS (D-PBS) and purified using iodixanol (Optiprep; AXS-1114542-250ML, Serumwerk Bernburg AG, Bernburg, Germany) and continuous-gradient ultracentrifugation. The viral solution was concentrated and formulated in D-PBS using a Vivaspin 20 column (VS2041 or VS2042; Sartorius AG, Göttingen, Germany). The genomic titers of the viral vectors were determined by PCR using Power SYBR Green PCR Master Mix (Thermo Fisher Scientific) using the primers 5′-CTGTTGGGCACTGACAATTC-3′ and 5′-GAAGG GACGTAGCAGAAGGA-3′ for the WPRE sequence, and the primers 5′-ACGCTGCTTTAATGCCTTTG-3′ and 5′-GAATTGTC AGTGCCCAACAG-3′ for the WPRE3 sequence. The expression plasmid was used as the standard.

Animals

The C57BL/6J mice used in this study were purchased from Jackson Laboratory Japan (Yokohama, Japan) and bred in the breeding colony at Gunma University Bioresource Center (Maebashi, Gunma, Japan). Ai14 reporter mice (Rosa-CAG-LSL-tdTomato; JAX: Ai14: [strain no. 007914]) were kindly provided by Dr. Daisuke Kohno (Gunma University). Ai75G mice were generated by inserting an NLS sequence before *tdTomato* in Ai14 mice. A sex-balanced group of mice were used for this study. All of the animal care and treatment procedures were performed in accordance with the Japanese Act on the Welfare and Management of Animals and the Guidelines for Proper Conduct of Animal Experiments issued by the Science Council of Japan. The experimental protocol was approved by the Institutional Committee of Gunma University (nos. 21–063 and 21–065). Every effort was made to minimize suffering and to reduce the number of animals used.

Generation of Ai75G mice

Ai75G mice were generated by inserting an NLS sequence before *tdTomato* in the Ai14 mice. The C57BL/6J and ICR mice were purchased from Charles River Laboratories (Yokohama, Japan). To prepare Ai14 zygotes, superovulation was induced in C57BL/6J female mice by injecting 5 U of pregnant mare serum gonadotropin (SEROTROPIN, ASKA Pharmaceutical, Tokyo, Japan), followed by the administration of 5 U of human chorionic gonadotropin (GONATROPIN, ASKA Pharmaceutical) 48 h later. After 17 h, MII oocytes were isolated from the oviduct, and *in vitro* fertilization was performed using the sperm derived from an Ai14 male mouse in CARD medium (Kyudo, Tosu, Saga, Japan). After 3 h of incubation, the zygotes were transferred to M16 medium (Sigma-Aldrich) supplemented with 1 mM EDTA/2Na. Genome editing by electroporation was performed as described previously.[25] Briefly, an electrode (LF501PT1-10; BEX, Tokyo, Japan) connected to a CUY21EDIT electroporator (BEX) was placed under a stereoscopic microscope. Ai14 zygotes were placed in a line in the electrode gap filled with 5 μL recombinant Cas9 protein (100 ng/μL; EnGen Cas9 NLS, New England Biolabs, Ipswich, MA), combined CRISPR RNA (crRNA)/trans-activating crRNA (3 μM, IDT, Coralville, IA), and donor DNA (7.6 μM, Ultramer DNA Oligo, IDT) in Opti-MEM I medium (Life Technologies, Carlsbad, CA). The target sequences used in this study were as follows: crRNA (5′-gcaacacgatcccgccacca-3′) and donor DNA (5′-ctccatgcgcaccttgaagcgcatgaactctttgatgacctcctcgcccttgctcaccatcacct ttctcttcttcttaggcatggtggcgggatcgtgttgcacttaacgcgtacaaggccggccgaattcg atctagcttgg-3′).

Electroporation was performed at 30 V (3 ms on + 97 ms off) using 7 electrical pulses. Genome-edited embryos that were developed to the 2-cell stage were transferred into the oviducts of pseudopregnant ICR females. To detect NLS insertion, genomic sequencing analysis was performed using tail tips from the offspring.

AAV injection through retro-orbital sinus

After deep anesthesia via an intraperitoneal injection of ketamine (100 mg/kg body weight) and xylazine (10 mg/kg body weight), 100 μL of the AAV solution was injected through the retro-orbital sinus for 20–30 s, using a 1-mL syringe with a 30G needle (08–277; Nipro, Osaka, Japan).

Stereotaxic injection of AAV5 into mouse primary motor cortex

After inducing deep anesthesia with ketamine/xylazine, the mice were positioned in a stereotactic frame and the skin covering the frontal bone was cut. The tip of a Hamilton syringe (33G) with a stereotaxic micromanipulator (SMM-100; Narishige, Tokyo, Japan) was inserted into the primary motor cortex (coordinates from bregma: anterior and posterior, +1.0 mm; midline, ±1.0 mm; dorsal and ventral, 0.8 mm). Viral solution (1×10^{12} vg/mL, 0.5 μL) was injected at a rate of 25 nL/min. After the injection, the needle remained in place for an additional 2 min and was withdrawn slowly.

Immunohistochemistry

Between 20 and 22 days after viral injection, deeply sedated mice were transcardially perfused with PBS (pH 7.4) and 4% paraformaldehyde in 0.1 M phosphate buffer (4% PFA/PB). Whole brains were immersed in 4% PFA/PB for 4–5 h at 4°C and cut sagittally into 30-μm (Figures 2, 3, 4, 5, 7, S2, S4, and S5) or 50-μm (Figures 1, 6, and S3) sections, using a microtome (Leica VT1200 S; Leica Microsystems, Wetzlar, Germany). Free-floating brain sections were blocked with PBS containing 2% normal donkey serum, 2% BSA, 0.05% Triton X-100, and 0.05% NaN_3 (blocking solution), and then incubated overnight at 4°C with primary antibodies. In the case of Figures 6 and S3, the primary antibodies were incubated overnight at room temperature. These primary antibodies for immunostaining were rabbit polyclonal anti-S100B (1:1,000; S100B-Rb-Af1000; Nittobo Medical, Tokyo, Japan), mouse monoclonal anti-NeuN (1:1,000; MAB377; Merck, Darmstadt, Germany), goat polyclonal anti-Olig2 (1:100; AF2418; R&D Systems, Minneapolis, MN), rat monoclonal anti-CD31 (1:100; 550274; BD Pharmingen, Franklin Lakes, NJ), and rat monoclonal anti-GFP (1:1,000; 04404-84; nacalai tesque, Kyoto, Japan). After rinsing several times with PBS containing Triton X-100 at room temperature, the slices were incubated with the following secondary antibodies overnight at 4°C in blocking solution: Alexa Fluor Plus 405 donkey anti-rabbit immunoglobulin G (IgG), Alexa Fluor Plus 488 donkey anti-goat IgG, Alexa Fluor Plus 488 donkey anti-rabbit IgG, Alexa Fluor Plus 488 donkey anti-rat IgG, Alexa Fluor Plus 555 donkey anti-goat IgG, Alexa Fluor Plus 647 donkey anti-rat IgG, and Alexa Fluor Plus 647 donkey anti-mouse IgG. As shown in Figures 6 and S3, secondary antibodies were incubated for 3 h at room temperature. Secondary antibodies were diluted 1:2,000. After washing using the procedure described above, the immunostained sections were mounted on glass slides with ProLong diamond antifade reagent (Thermo Fisher Scientific).

Imaging analysis

Fluorescent images of the brain sections were acquired using a laser-scanning confocal microscope (LSM 800; Carl Zeiss, Oberkochen, Germany) with a 10× objective, and z stack images of different focal planes were generated. GFP images of the whole brain were acquired using a fluorescence stereoscopic microscope (VB-7010; Keyence, Osaka, Japan). The GFP fluorescence intensity of the whole brain was measured using ImageJ software (https://imagej.net/software/fiji/).[26] The outline of the whole brain was traced, and the fluorescence intensity in the enclosed areas was measured accordingly.

Measurement of specificities and efficiencies for astrocyte or neuronal transduction

To determine the specificity in astrocytes or neurons and the efficiency in these cell types, the fluorescent images (639.0 × 639.0 μm) were acquired in two different regions of the cerebral cortex (M1 or M2) of each sagittal brain section using a confocal laser-scanning microscope with a 10× objective. The S100B^{+} (n > 300), tdTomato^{+} (n > 180), and NeuN^{+} (n > 1,200) images of each mouse brain were automatically counted using ImageJ Fiji. In Figure 7, a total of at least 30 tdTomato^{+} cells in each coronal brain section were counted manually. The specificity for astrocyte transduction was calculated by dividing the number of cells that were double positive for S100B and tdTomato by the number of tdTomato-expressing cells. The efficiency of astrocyte transduction was calculated as the number of cells that were double positive for S100B and tdTomato divided by the number of S100B-immunolabeled cells. Similarly, the specificity and efficiency of neuronal transduction were calculated using NeuN labeling instead of S100B labeling. In Figure S3, the transduction specificities for astrocytes, neurons, and oligodendrocytes were determined by dividing the number of double-positive cells (GFP and cell-type marker) by the number of GFP^{+} cells.

Statistical analysis

GraphPad Prism (version 9; GraphPad Software, San Diego, CA) was used for the statistical analysis and to generate schematic images. The statistical methods are shown in each figure legend. The data are expressed as mean ± SD. A $p < 0.05$ was considered statistically significant.

DATA AND CODE AVAILABILITY

The datasets and programs generated for this study are available from the corresponding author upon request.

SUPPLEMENTAL INFORMATION

Supplemental information can be found online at https://doi.org/10.1016/j.omtm.2024.101185.

ACKNOWLEDGMENTS

The authors thank Asako Ohnishi, Ayako Sugimoto, and Nobue McCullough for AAV vector production and Junko Sugiyama for raising the mice. This research was partially supported by the Program

for Brain Mapping by Integrated Neurotechnologies for Disease Studies (Brain/MINDS) from the Japan Agency for Medical Research and Development (AMED) under grant nos. JP19dm0207057, JP20dm0207057, and JP21dm0207111, and JSPS KAKENHI (grant nos. 23H02791, 19K06899, and 22K06454) and the Platform Project for Supporting Drug Discovery and Life Science Research (Basis for Supporting Innovative Drug Discovery and Life Science Research) from AMED under grant JP21am0101120 (support No. 2953).

AUTHOR CONTRIBUTIONS

H.H. supervised the study. H.K., A.K., Y.M., and H.H. designed the experiments. H.K., A.K., Y.M., Y.S., M.K., R.A., S. Tsutsumi, and S. Togai performed the experiments. R.K., I.H., and T.H. produced the Ai75G mice. H.K. and A.K. prepared the original drafts of the manuscript. All of the authors read and approved the final version of the manuscript.

DECLARATION OF INTERESTS

The authors declare no competing interests.

REFERENCES

1. Hoshino, C., Konno, A., Hosoi, N., Kaneko, R., Mukai, R., Nakai, J., and Hirai, H. (2021). GABAergic neuron-specific whole-brain transduction by AAV-PHP.B incorporated with a new GAD65 promoter. Mol. Brain *14*, 33.
2. Nitta, K., Matsuzaki, Y., Konno, A., and Hirai, H. (2017). Minimal Purkinje Cell-Specific PCP2/L7 Promoter Virally Available for Rodents and Non-human Primates. Mol. Ther. Methods Clin. Dev. *6*, 159–170.
3. Shinohara, Y., Ohtani, T., Konno, A., and Hirai, H. (2017). Viral Vector-Based Evaluation of Regulatory Regions in the Neuron-Specific Enolase (NSE) Promoter in Mouse Cerebellum In Vivo. Cerebellum *16*, 913–922.
4. Chan, K.Y., Jang, M.J., Yoo, B.B., Greenbaum, A., Ravi, N., Wu, W.L., Sánchez-Guardado, L., Lois, C., Mazmanian, S.K., Deverman, B.E., and Gradinaru, V. (2017). Engineered AAVs for efficient noninvasive gene delivery to the central and peripheral nervous systems. Nat. Neurosci. *20*, 1172–1179.
5. Deverman, B.E., Pravdo, P.L., Simpson, B.P., Kumar, S.R., Chan, K.Y., Banerjee, A., Wu, W.L., Yang, B., Huber, N., Pasca, S.P., and Gradinaru, V. (2016). Cre-dependent selection yields AAV variants for widespread gene transfer to the adult brain. Nat. Biotechnol. *34*, 204–209.
6. Hanlon, K.S., Meltzer, J.C., Buzhdygan, T., Cheng, M.J., Sena-Esteves, M., Bennett, R.E., Sullivan, T.P., Razmpour, R., Gong, Y., Ng, C., et al. (2019). Selection of an Efficient AAV Vector for Robust CNS Transgene Expression. Mol. Ther. Methods Clin. Dev. *15*, 320–332.
7. Brenner, M., Kisseberth, W.C., Su, Y., Besnard, F., and Messing, A. (1994). GFAP promoter directs astrocyte-specific expression in transgenic mice. J. Neurosci. *14*, 1030–1037.
8. Lee, Y., Messing, A., Su, M., and Brenner, M. (2008). GFAP promoter elements required for region-specific and astrocyte-specific expression. Glia *56*, 481–493.
9. Dirren, E., Towne, C.L., Setola, V., Redmond, D.E., Jr., Schneider, B.L., and Aebischer, P. (2014). Intracerebroventricular injection of adeno-associated virus 6 and 9 vectors for cell type-specific transgene expression in the spinal cord. Hum. Gene Ther. *25*, 109–120.
10. Heffernan, K.S., Rahman, K., Smith, Y., and Galvan, A. (2022). Characterization of the GfaABC1D promoter to selectively target astrocytes in the rhesus macaque brain. J. Neurosci. Methods *372*, 109530.
11. Vagner, T., Dvorzhak, A., Wójtowicz, A.M., Harms, C., and Grantyn, R. (2016). Systemic application of AAV vectors targeting GFAP-expressing astrocytes in Z-Q175-KI Huntington's disease mice. Mol. Cell. Neurosci. *77*, 76–86.
12. Shinohara, Y., Konno, A., Nitta, K., Matsuzaki, Y., Yasui, H., Suwa, J., Hiromura, K., and Hirai, H. (2019). Effects of Neutralizing Antibody Production on AAV-PHP.B-Mediated Transduction of the Mouse Central Nervous System. Mol. Neurobiol. *56*, 4203–4214.
13. Radhiyanti, P.T., Konno, A., Matsuzaki, Y., and Hirai, H. (2021). Comparative study of neuron-specific promoters in mouse brain transduced by intravenously administered AAV-PHP.eB. Neurosci. Lett. *756*, 135956.
14. Kawashima, T., Kitamura, K., Suzuki, K., Nonaka, M., Kamijo, S., Takemoto-Kimura, S., Kano, M., Okuno, H., Ohki, K., and Bito, H. (2013). Functional labeling of neurons and their projections using the synthetic activity-dependent promoter E-SARE. Nat. Methods *10*, 889–895.
15. Li, X., Zhao, X., Fang, Y., Jiang, X., Duong, T., Fan, C., Huang, C.C., and Kain, S.R. (1998). Generation of destabilized green fluorescent protein as a transcription reporter. J. Biol. Chem. *273*, 34970–34975.
16. Ringrose, L., Lounnas, V., Ehrlich, L., Buchholz, F., Wade, R., and Stewart, A.F. (1998). Comparative kinetic analysis of FLP and cre recombinases: mathematical models for DNA binding and recombination. J. Mol. Biol. *284*, 363–384.
17. Rechsteiner, M., and Rogers, S.W. (1996). PEST sequences and regulation by proteolysis. Trends Biochem. Sci. *21*, 267–271.
18. Ortinski, P.I., Dong, J., Mungenast, A., Yue, C., Takano, H., Watson, D.J., Haydon, P.G., and Coulter, D.A. (2010). Selective induction of astrocytic gliosis generates deficits in neuronal inhibition. Nat. Neurosci. *13*, 584–591.
19. Ghosh, K., and Van Duyne, G.D. (2002). Cre-loxP biochemistry. Methods (San Diego, Calif.) *28*, 374–383.
20. Gao, Y., Fang, K., Yan, Z., Zhang, H., Geng, G., Wu, W., Xu, D., Zhang, H., Zhong, N., Wang, Q., et al. (2022). Develop an efficient and specific AAV-based labeling system for Muller glia in mice. Sci. Rep. *12*, 22410.
21. Park, Y.M., Chun, H., Shin, J.I., and Lee, C.J. (2018). Astrocyte Specificity and Coverage of hGFAP-CreERT2 [Tg(GFAP-Cre/ERT2)13Kdmc] Mouse Line in Various Brain Regions. Exp. Neurobiol. *27*, 508–525.
22. Choi, J.H., Yu, N.K., Baek, G.C., Bakes, J., Seo, D., Nam, H.J., Baek, S.H., Lim, C.S., Lee, Y.S., and Kaang, B.K. (2014). Optimization of AAV expression cassettes to improve packaging capacity and transgene expression in neurons. Mol. Brain *7*, 17.
23. Yeo, S., Bandyopadhyay, S., Messing, A., and Brenner, M. (2013). Transgenic analysis of GFAP promoter elements. Glia *61*, 1488–1499.
24. Konno, A., and Hirai, H. (2020). Efficient whole brain transduction by systemic infusion of minimally purified AAV-PHP.eB. J. Neurosci. Methods *346*, 108914.
25. Horii, T., Kobayashi, R., and Hatada, I. (2023). Generation of Floxed Mice by Sequential Electroporation. Methods Mol. Biol. *2637*, 135–147.
26. Schindelin, J., Arganda-Carreras, I., Frise, E., Kaynig, V., Longair, M., Pietzsch, T., Preibisch, S., Rueden, C., Saalfeld, S., Schmid, B., et al. (2012). Fiji: an open-source platform for biological-image analysis. Nat. Methods *9*, 676–682.

OMTM, Volume *32*

Supplemental information

Improving cell-specific recombination using AAV vectors in the murine CNS by capsid and expression cassette optimization

Hayato Kawabata, Ayumu Konno, Yasunori Matsuzaki, Yumika Sato, Mika Kawachi, Ryo Aoki, Saki Tsutsumi, Shota Togai, Ryosuke Kobayashi, Takuro Horii, Izuho Hatada, and Hirokazu Hirai

mGfaABC1D promoter: 582bp

5’- AACAAATACCATGTCGCTGGTATGGAGTATAGGCTGTTGCTATGACAGGAACT
CAGGGGTCTTAACTGGCTTGAGCGCTGGGAGGGGGCAAGCAGCCAGGCCT
TGTCTGTAAGCTGAAGACCTGGCAGTGCTGAGCTGGTCAGCCCCCAGGACC
TCCTTTTGTGCCCACGAGTGACTCACCTTGGCATAGACATAATGGTCAGGGG
TGGGCACGCAGCCTGCTTCCCGCTGTGCTCCAGGCCTCCTTCGATGCTTTC
CGAGAAGTCTATTGAGCTGGGAGCTTGTACTGCACCCGGGGCTGACATCCT
GGCATCCTGGGATAAAAGCAGCCCACGGGGCTGCCCTTGCCATATGCCTCA
CTGGCGGCAGAGAACAAGGCTCTATTCAGCGAGTACCCTGGGGAGTTCTCC
CCCTAGCTGGGCTGGGGCCCAGCTCCACCCCCTCAGGCTATTCAATGGGG
GTGCTTCCAGGAAGTCAGGGGCAGATTTAGTCCAACCCGTTCCTCCATAAAG
GCCCTGACATCCCAGGAGCCAGCAGAGGCAGGGCAGGTTGGAGCGGAGA
CGCATCACCTCTGCGCGCCGC -3’

Figure S1. Sequence of astrocyte-specific mouse GfaABC1D promoter used in this study.

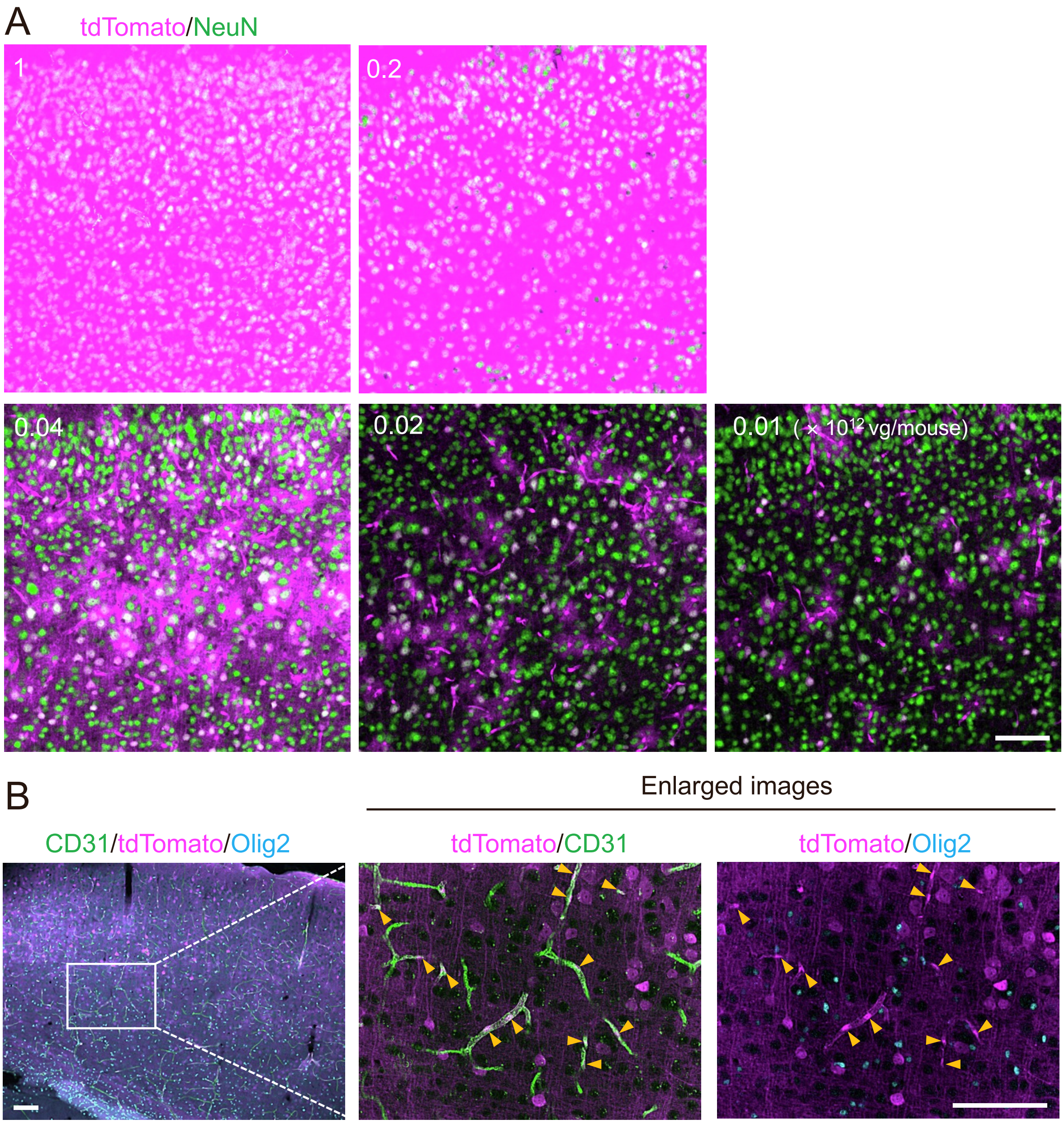

Figure S2. Non-specific cellular transduction in Ai14 floxed mouse brain by intravenously administered PHP.eB expressing Cre using mGfaABC1D promoter. Immunohistochemistry of cerebral cortex three weeks after administration of AAV injection. Mice received injection of AAV vectors serially diluted from 1×10^{12} vg/mouse to 1×10^{10} vg/mouse as shown on the upper left corner of each panel. (**A**) Numerous tdTomato-expressing cells immunolabeled for NSE, a neuron marker. (**B**) Expression of tdTomato in CD31-positive brain microvascular endothelial cells (orange arrowheads). Sagittal brain sections were

immunolabeled for CD31 (green), a marker of brain microvascular endothelial cell and Olig2 (light blue), and a marker of oligodendrocyte. Scale bars; 100 μm.

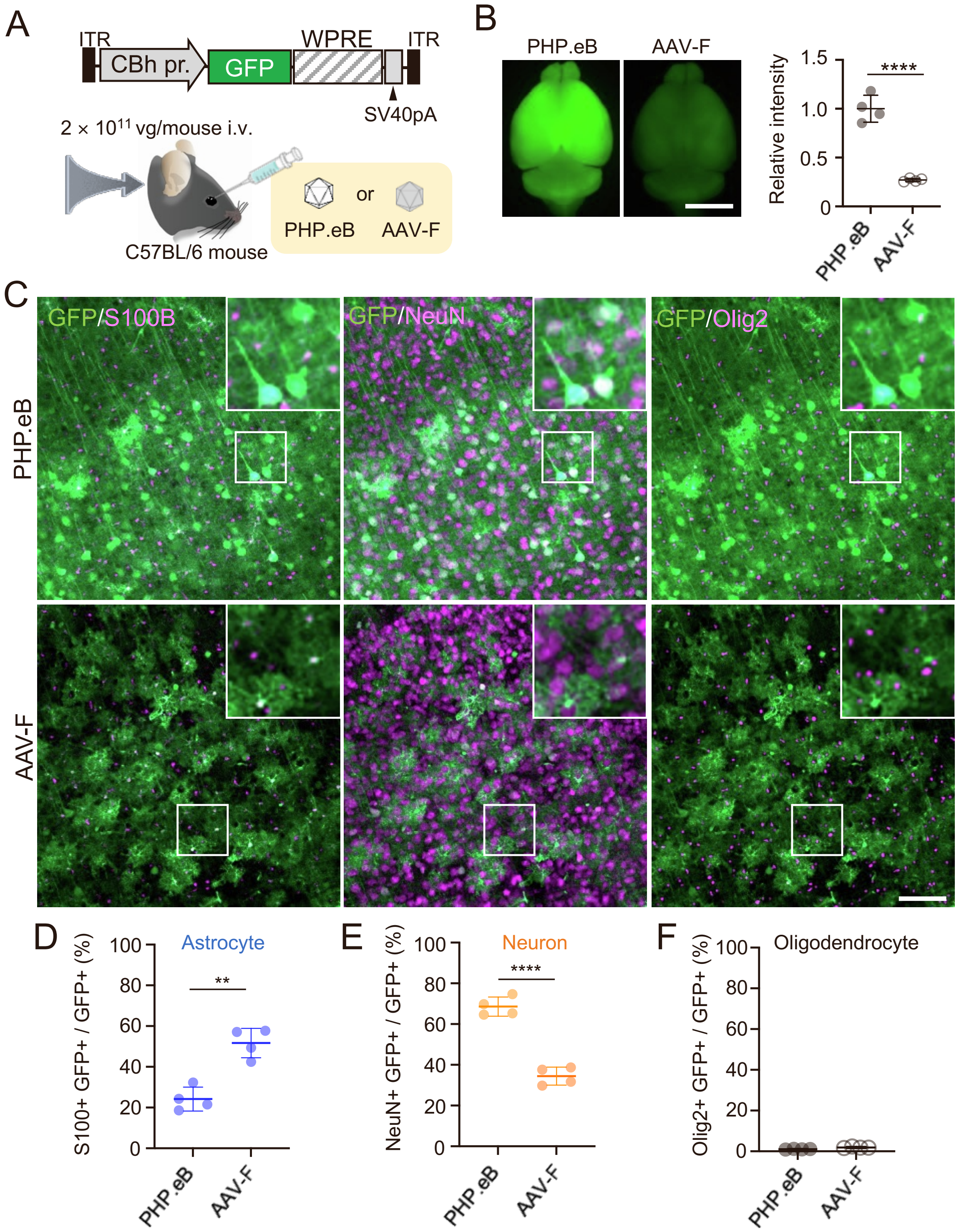

Figure S3. Higher astrocyte-tropic nature of AAV-F over PHP.eB. (**A**) Schematic diagram showing experimental procedure. PHP.eB or AAV-F capsid vectors expressing green fluorescence protein (GFP) under control of ubiquitously active chicken β-actin hybrid

(CBh) promoter were injected into wild-type C57BL/6 mice through orbital plexus. (**B**) Significantly higher fluorescence intensity in PHP.eB-treated mouse brain than in AAV-F-treated brain. GFP fluorescence intensity of whole brains was measured three weeks after AAV injection. Data (average ± SD) were obtained from four mice and value from each mouse was plotted. ****P<0.0001 by unpaired student's *t*-test. Scale bar; 5 mm. (**C**) Triple immunostaining of brain sagittal sections for S100B, NeuN, and Olig2, an oligodendrocyte marker, three weeks after AAV injection. Fluorescence images of primary motor cortex from PHP.eB-treated (upper panels) and AAV-F-treated (lower panels) mice were presented, in which square regions were enlarged and shown in upper right corners. Scale bar; 100 μm. (**D–F**) Graph showing specificity for transduction of astrocytes (**D**), neurons, (**E**) and oligodendrocytes (**F**) in mice treated with PHP.eB or AAV-F vectors. Data (average ± SD) were obtained from four mice and value from each mouse was plotted. **P<0.01 and ***P<0.001 by unpaired student's *t*-test. ITR; inverted terminal repeat, WPRE; woodchuck hepatitis virus post-transcriptional regulatory element, SV40pA; simian virus 40 polyadenylation signal.

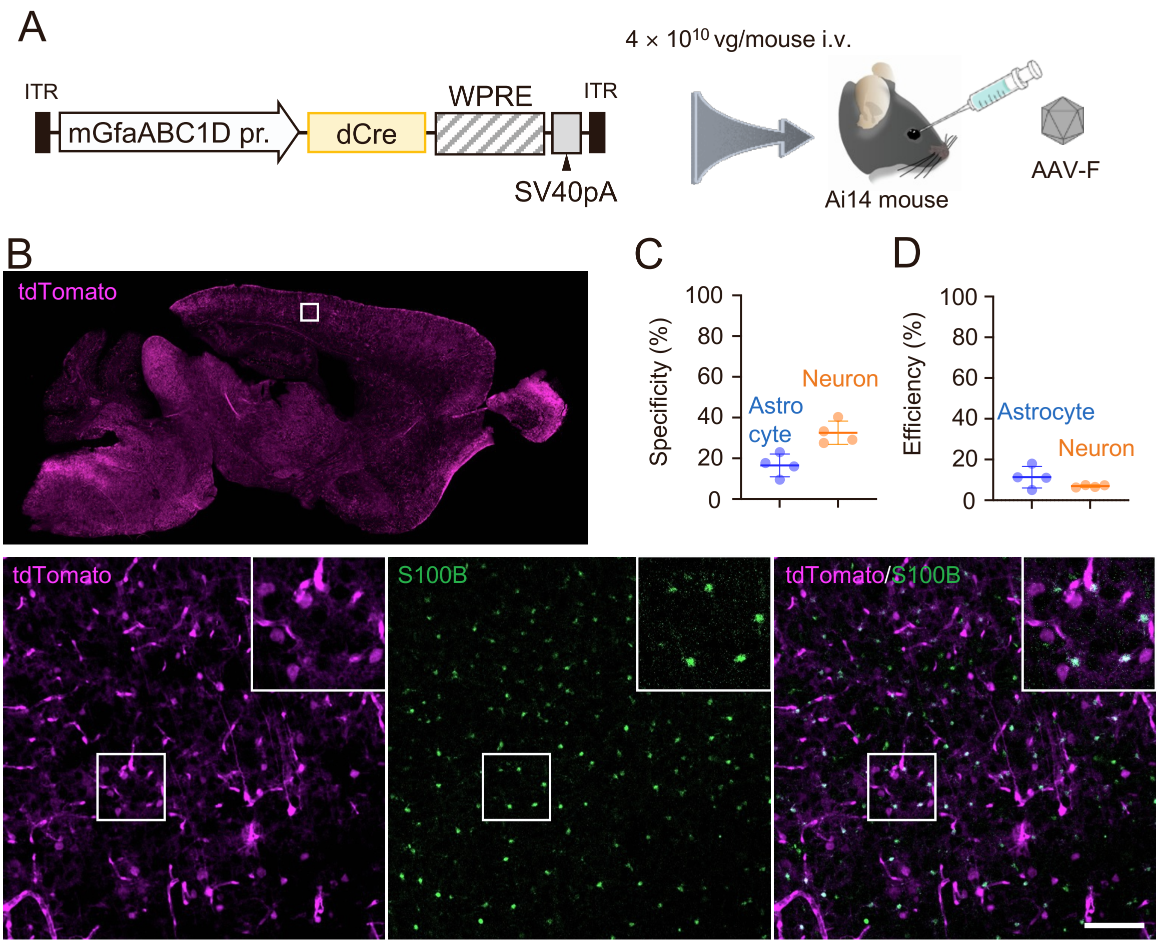

Figure S4. Non-specific recombination in Ai14 mouse brain by AAV-F- mGfaABC1D promoter-dCre. (**A**) Schematic diagram showing experimental procedure. Ai14 mice received intravenous injection of AAV-F-mGfaABC1D promoter-dCre. (**B**) Double immunostaining of brain sagittal sections for S100B (green) and NeuN (not shown) three weeks after AAV injection, showing that most of tdTomato-expressing cells were not immunolabeled for S100B. Scale bar; 100 µm. (**C**, **D**) Graph showing specificity (**C**) and efficiency (**D**) for astrocyte and neuronal transduction. Data (average ± SD) were obtained from four mice and value from each mouse was plotted. The specificity and efficiency for astrocytes are presented in Table S1.

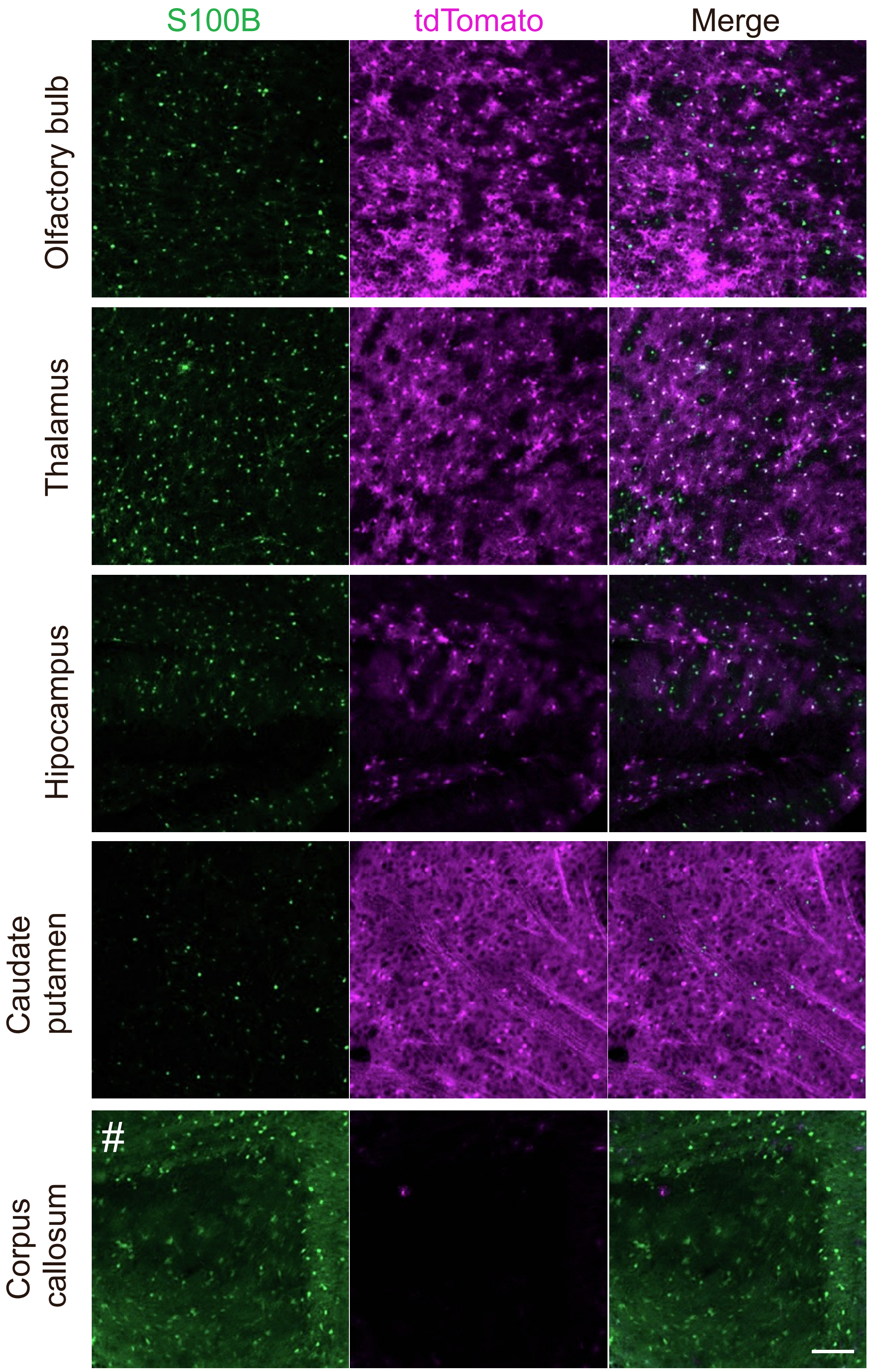

Figure S5. Recombination profiles of various regions in Ai14 mouse treated with AAV-F/FlpO-dCre. Ai14 mice were treated with AAV-F/FlpO-dCre as depicted in Fig. 5A and

examined three weeks after the injection. Fluorescence images of S100B immunolabeling (green, left panels), native tdTomato (magenta, middle panels) and merged images (right panels) from various brain regions. Fluorescent images were obtained using the same parameters except for S100B immunostaining of corpus callosum (#, bottom left panel), which was enhanced because the fluorescence was too weak. Scale bar; 100 μm. The specificity and efficiency of astrocyte recombination in the mentioned brain areas are summarized in Table S2.

Table S1. Summary of the specificity and efficiency of astrocyte transduction in the cerebral cortex by intravenous infusion of PHP.eB or AAV-F capsid vectors. These AAV vectors expressed tdTomato, Cre, FlpO, and dCre under control of mGfaABC1D promoter. Specificity for astrocyte transduction was calculated by dividing number of tdTomato and S100B double-positive cells by number of tdTomato-positive cells. Transduction efficiency was calculated by dividing number of tdTomato and S100B double-positive cells by number of S100B-positive cells. Data (average ± SD) were obtained from four mice.

AAV		Mouse	Dose (vg/mouse)	Astrocyte	
Genome	Capsid			Specificity (%)	Efficiency (%)
mGfaABC1D-tdTomato	PHP.eB	WT	1.0E+12	90.6 ± 4.1	13.2 ± 8.2
mGfaABC1D-Cre	PHP.eB	Ai14	4.0E+10	9.3 ± 3.5	10.8 ± 7.8
mGfaABC1D-Cre	AAV-F	Ai14	4.0E+10	33.6 ± 5.6	17.6 ± 3.6
mGfaABC1D-dCre	AAV-F	Ai14	4.0E+10	16.6 ± 4.8	11.3 ± 4.6
mGfaABC1D-FlpO<br>mGfaABC1D-dCre	PHP.eB	Ai14	5.0E+11<br>5.0E+11	49.9 ± 10.9	43.3 ± 6.8
mGfaABC1D-FlpO<br>mGfaABC1D-dCre	AAV-F	Ai14	5.0E+11<br>5.0E+11	92.6 ± 1.4	42.8 ± 9.7

Table S2. Summary of the specificity and efficiency of astrocyte transduction in different brain regions from Ai14 mice treated with AAV-F/FlpO-dCre. Data (average ± SD) were obtained from four–five mice except for the data on the corpus callosum (n = 2 mice). N.D., not determined.

	Astrocyte	
Region	Specificity (%)	Efficiency (%)
Olfactory bulb	62.0±14.1	48.7±16.3
Thalamus	81.8±15.1	32.2±10.0
Hippocampus	88.4±6.9	22.3±5.7
Caudate putamen	37.3±21.4	8.7±5.9
Corpus callosum	N.D.	N.D.
